# Estimating the heterogeneous health and well‐being returns to social participation

**DOI:** 10.1002/hec.4695

**Published:** 2023-05-05

**Authors:** Anna Wilding, Luke Munford, Matt Sutton

**Affiliations:** ^1^ Health Organisation, Policy and Economics The University of Manchester Manchester UK; ^2^ Centre for Health Economics Monash University Melbourne Australia

**Keywords:** community assets, marginal treatment effects, social determinants of health, social participation, well‐being

## Abstract

Social participation is defined as an individual's involvement in activities that provide connections with others in society. Past research has demonstrated links between social participation, improved health and well‐being, and reduced social isolation, but has been restricted to older people and has not investigated heterogeneity. Using cross‐sectional data from the UK's Community Life Survey (2013–2019; *N* = 50,006), we estimated returns to social participation in the adult population. We included availability of community assets as instruments in a marginal treatment effects model, which allows treatment effects to be heterogeneous and examines whether the effects vary by propensity to participate. Social participation was linked to reduced loneliness and improved health (−0.96 and 0.40 points respectively on 1–5 scales) and increased life satisfaction and happiness (2.17 and 2.03 points respectively on 0–10 scales). These effects were larger for those on low income, with lower education attainment, and who live alone or with no children. We also found negative selection, indicating those less likely to participate have higher health and well‐being returns. Future interventions could focus on increasing community asset infrastructure and encouraging social participation for those with lower socio‐economic status.

## 1 INTRODUCTION

Social participation is defined as an individual's involvement in activities that provide connections with others in the society or community (Aroogh & Shahboulaghi, 2020; Levasseur et al., 2010). This can be achieved by engaging in a specific activity or volunteering (Richard et al., 2013). Over the past decade, there has been renewed interest amongst health and local government agencies in the United Kingdom (UK) in policies to encourage social participation. Most recently, the National Health Service (NHS) England has introduced “social prescribing” programmes in family practitioner practices (Howarth et al., 2020). These social prescribing schemes involve patients being prescribed for attending social groups on top of, or as an alternative to, medical therapeutics. Slightly earlier, the Local Government Association issued guidance to encourage community engagement, focusing on interventions to increase social participation (Local Government Association, 2017). Both policy initiatives aim to improve the health and well‐being of participants.

To enable social participation, there needs to be social infrastructure available since this can act as a facilitator or barrier (Roskruge et al., 2012). There are two critical components of access: proximity to the assets; and whether an individual can walk there to remove transportation barriers (Sáinz‐Ruiz et al., 2021). Within England, these social infrastructures have been called “Community Assets” (Brown & Barber, 2012). These amenities include: leisure facilities, libraries, parks, community centers and churches. They are predominately publicly‐owned and also include institutions such as schools and healthcare centers such as family practices. In addition, there are “Assets of Community Value,” a scheme whereby individuals or groups can nominate derelict public‐owned buildings or spaces for community ownership, giving them access to resources from a “Community Renewal Fund” to rejuvenate and improve assets (Department for Levelling Up, Housing and Communities, 2021).

Previous research has shown that availability of some community assets encourages social participation, particularly sports facilities, youth clubs, community centers and places of worship (Wilding et al., 2022). Using a large, national cross‐sectional survey of adults in England (the *Community Life Survey*), this study showed that individuals who reported these community assets were available locally, were more likely to report social participation. Furthermore, it showed that individuals who engaged in social participation had different demographic and socioeconomic characteristics. For example, individuals who were female, of Black, Asian or minority ethnicity, homeowners, had higher household income, and resided in less deprived areas were more likely to participate.

Engagement in groups and networks that use these assets and facilities has been predicted to reduce health and well‐being inequalities (Foot & Hopkins, 2010). Social functioning is included directly as a component of many measures of well‐being, for example, the Short Form health instrument contains a social interactions component (Ware, 2002) and the General Health Questionnaire includes a question on the enjoyment of activities (Goldberg & Hillier, 1979). Previous research on health consequences has shown that social participation through group membership leads to improved health outcomes (Munford, Panagioti, et al., 2020; Munford, Wilding, et al., 2020) and reduced mortality (Glass et al., 1999; Steffens et al., 2016) in older populations.

Given the wider age range targeted by current social participation policies, evidence is required on a more general population rather than just the older populations considered in previous studies. Moreover, although past studies have demonstrated significant associations with outcomes, other studies have raised endogeneity concerns due to reverse causality (Ichida et al., 2013; Tomioka et al., 2017) and selection bias (Dawson‐Townsend, 2019).

In this paper, we aim to estimate causal effects of social participation on health and well‐being amongst the general adult population. The literature has highlighted the different characteristics of individuals engaging in social participation, likely due, in part, to variations in barriers to entry. For example, individuals living in more deprived areas may face higher travel costs to reach community assets. In addition, there is likely to be unobserved heterogeneity between individuals in their willingness to engage in social participation. Consequently, we focus on examining whether the (potential) benefits of social participation are homogenous, as previous studies have assumed.

To overcome the endogeneity concerns when estimating the effect of social participation on outcomes, we use an instrumental variable specification. We use the local availability of community asset infrastructure to predict social participation in the first‐stage equation. We undertake a range of supplementary analyses to check that community asset infrastructure does not directly affect health and well‐being, only through enacting social participation. Given the differing participation levels across population characteristics and the potential for selection bias, we use a marginal treatment effects model (Heckman & Vytlacil, 2007) to estimate heterogeneous treatment effects in this instrumental variable specification.

## 2 DATA

We use data from the Community Life Survey as this dataset contains information on community asset availability and social participation. Other datasets, such as the “Taking Part” (Department for Culture, Media and Sport, 2021), “Active Lives Survey” (Sport England, 2020) and “Understanding Society” (University of Essex, 2022), do not contain this information on community asset infrastructure. The Community Life Survey is a series of random stratified cross‐sectional samples of English individuals aged over 16 years (Department for Culture, Media and Sport, 2019). We combine 2013/14 to 2020/21 to form a pooled cross‐section dataset. The individual‐level dataset contains information on social participation status, health and well‐being, community asset availability (self‐perceived), socio‐economic status, and a wealth of demographic information.

### 2.1 Outcomes

We use health, well‐being and loneliness measures as outcomes. For health, we use self‐assessed health for which individuals are asked, “In general, would you say your health is…” with options from Excellent (assigned a score of 5), Very Good, Good, Fair, and Poor (assigned a score of 1). This self‐reported health measure is a reliable proxy for more objective health measures such as hospitalizations, family practitioner use and prescription costs (Doiron et al., 2015; Hanlon et al., 2007).

To measure well‐being, we use the ONS4 measures (Tinkler, 2015). These are four questions relating to personal well‐being, summarized in Table 1. Each question is answered on an 11‐point scale ranging from 0 (not at all) to 10 (completely). These scores have been validated for use within evaluations of social prescribing schemes (What Works Wellbeing, 2020). We reverse coded the responses to the anxiety measure for ease of interpretation and comparison with the other measures within the ONS4.

**TABLE 1:** ONS4 measures of well‐being.

Measure	Question	Responses
Life satisfaction	Overall, how satisfied are you with your life nowadays?	0 (not at all) to 10 (completely)
Worthwhileness	Overall, to what extent do you feel that the things you do in your life are worthwhile?	0 (not at all) to 10 (completely)
Happiness	Overall, how happy did you feel yesterday?	0 (not at all) to 10 (completely)
Anxiety	How anxious did you feel yesterday?	0 (completely anxious) to 10 (not at all anxious)

*Source*: Office for National Statistics.

We also examine loneliness, as previous research has shown links between increased social participation and reduced loneliness (Masi et al., 2011). Loneliness is detrimental to physical and mental health, particularly in the older population (Gerst‐Emerson & Jayawardhana, 2015). The survey asks individuals, *“How often do you feel lonely?”* with the following responses: (1) Often/Always, (2) Some of the Time, (3) Occasionally, (4) Hardly Ever, and (5) Never. This is a measure of the frequency of loneliness and has been widely used in UK (Victor & Yang, 2012) and European studies (Yang & Victor, 2011). However, the measure could under‐report actual levels of loneliness due to stigma (Pinquart & Sorensen, 2001). The more accurate 11‐item scale was not asked within the survey, but cross‐validation has shown a high correlation between the two (Victor et al., 2005).

### 2.2 Social participation

The survey asks individuals about their past year's involvement in groups, clubs and organizations. Based on the definition of Levasseur et al. (p. 2148), “*a person's involvement in activities that provide interaction with others in society or the community”,* we use responses to this survey question to measure social participation. The list of possible groups is contained in Table 2, which shows a wide range of ways individuals can interact with their local community.

**TABLE 2:** List of groups, clubs and organizations included in the Community Life Survey.

Option	Group	Option	Group
1	Sport/exercise	9	Safety & first aid
2	Local community	10	Education for adults
3	Religion	11	Children's activities
4	Hobbies & arts	12	The elderly
5	Children's education	13	Justice & human rights
6	Citizens	14	Politics
7	Environment	15	Health & social welfare
8	Safety & first aid		

*Source*: Community Life Survey.

From this question, we create a binary measure of social participation. If an individual responds by participating in at least one of the groups from Table 2, then an individual is defined as socially participating. This allows the measure to encapsulate a broad range of interests.

### 2.3 Community assets

The surveys also request information on the availability of local community assets with the following question: *“Which of these are located within a 15–20‐min walk from your home?”* for the types of assets listed in Table 3. We created binary variables from this question for each community asset type. These variables equal one if available and zero otherwise.

**TABLE 3:** List of community assets.

Option	Asset type	Option	Asset type
1	General/grocery shop	8	Health center/GP
2	Pub	9	Chemist
3	Park	10	Post office
4	Library	11	Primary school
5	Community center/hall	12	Secondary school
6	Sports center/club	13	Church/place of worship
7	Youth center/club	14	Public transport links

*Source*: Community Life Survey.

### 2.4 Covariates

We include a range of additional covariates to control for factors affecting an individual's health and well‐being. The survey captures a range of determinants of health, which can be split into individual‐, household‐ and area‐level characteristics, outlined in Table A1. Individual‐level characteristics include gender, age, ethnicity, marital status and education level. Household‐level characteristics include the logarithmic value of deflated and equivalised household income,^1^ household composition and housing tenure. Area‐level characteristics include the quintile of the Index of Multiple Deprivation 2015 (Noble et al., 2006), rurality, and the government office region. We additionally control for macroeconomic fluctuations and time trends common to all individuals within a given year by including survey wave fixed effects.

## 3 METHODS

Several papers have highlighted concerns about reverse causality (Ichida et al., 2013; Tomioka et al., 2017) and self‐selection (Dawson‐Townsend, 2019) when evaluating social participation. Reverse causality may occur because those with “better” health and well‐being are more able to participate socially. Self‐selection may arise if those who socially participate have different unmeasured characteristics than those who do not. This could lead to treatment effects not being externally valid due to not representing the general population, only the sub‐sample of those with characteristics of participants. To overcome these potential issues, we use the marginal treatment effects model developed by Heckman and Vytlacil (2007) with local instrument variables. This enables the use of a standard IV approach while estimating outcomes across the propensity scores.

The estimation of this model involves three stages. The first is a non‐linear estimation of social participation on covariates and some instrumental variables. The second stage consists of estimating returns to outcomes of social participation (treatment effects) using the predicted estimates of the endogenous variable from the first stage. The final step involves estimating the treatment effects across quantiles of the propensity scores of social participation from the first stage.

We adjust all models using population weights provided by the Community Life Survey. This ensures that our estimates are adjusted to the general population's characteristics to enhance the findings' external validity. Additionally, we use robust standard errors since the error terms may not be homoscedastic.

### 3.1 Instrumental variables

To be valid, instrumental variables must be relevant and meet both the exclusion and monotonicity criteria (Angrist & Imbens, 1995). We selected the instruments from the measures of community assets availability in Table 3. The instruments should only affect health, well‐being and loneliness through their effect on social participation. Violations to exclusion criteria can occur either through a direct relationship between the instrument and the outcomes or because both are correlated with an unobserved factor.

Based on previous research using the Community Life Survey (Wilding et al., 2022), we eliminated the following community assets because they do not predict social participation rates and therefore are not relevant: pubs, parks, public transport links, post offices, secondary schools, libraries, general shops, primary schools, chemists and GP/health centers. Therefore, we excluded these from the list of potential instruments.

The remaining four statistically‐relevant potential instruments are: youth centers, sports centers, community centers, and places of worship. We do not consider youth centers as a potential instrument, because they are not social participation hubs for our adult sample. We omit sports centers in the main specification because they may be located in areas where owners expected there to be more active and healthy populations (Hillsdon et al., 2007). Unmeasured health attitudes and abilities to undertake sports are likely to affect the location of sports centers and be correlated with health and well‐being outcomes. This would make sports center availability non‐excludable. In a supplementary analysis, we examine this possibility empirically.

In the main analysis, we select community centers and places of worship as instruments for social participation. The locations of these facilities are not based on expectations about the local population's health. Their sites are often historical to serve local communities (Aiken et al., 2016), and the motivation for their construction is for community cohesion (Brown & Barber, 2012; Mulkeen, 2019). Planning regulations for new housing developments require that they consider community infrastructure (Mulkeen, 2019), with the purpose of offering equality of access and fostering cohesion rather than being based on a pre‐existing absence or abundance of it. This weakens the link between community centers and pre‐existing cohesion levels which could be linked to outcomes. They are hubs for a broad range of groups and activities, and their multi‐purpose use makes them relevant for individuals of all ages.

Similarly, places of worship can be used for a broad range of (not necessarily religious) types of social participation. Many of the Church of England places of worship in England were built before World War II, and additional structures were built after the war to handle the displacement of individuals and new towns (Mulkeen, 2019). There was special legislation for the new towns to have a specific area for a Church to be built with reduced building costs. This weakens the potential deprivation level link due to reducing financial barriers to availability. England is a multi‐faith country, and places of worship encapsulates different religions. We hypothesize their availability will be based on the faith needs of the community rather than their health needs.

We include all the assets not used as instruments as covariates since they may be linked to outcomes, and including them will improve the precision of the estimates. For example, those with better availability of healthcare assets are likely to have improved health (Gravelle et al., 2008). There are also links between access to parks and green spaces and mental health (Wood et al., 2017). The availability of pubs and therefore access to alcohol has detrimental effects on both mental and physical health (Topiwala et al., 2021). Public transport links reflect rurality and remoteness, which are also linked to outcomes. Primary schools and secondary schools availability reflect the possibility of selective migration into school catchment areas (Croft, 2004) and the links between socioeconomic status and the quality of schools (Ball, 1993). The availability of pharmacies within the UK has been linked to deprivation which has also been linked to health and well‐being (Todd et al., 2015).

### 3.2 Marginal treatment effects model

The underlying model is derived from the Roy model for selection (Roy, 1951), with the addition of treatment effects that vary by propensity scores (Heckman & Vytlacil, 2007). The model setup is as follows:

(1) $$\begin{array}{cc} Y_{j}=\mu_{j}\left(X\right)+U_{j} & for\;j=0,1 \end{array}$$

(2) $$Y=DY_{1}+\left(1-D\right)Y_{0}$$

(3) $$\begin{array}{cc} D=1\left\{\mu_{D}\left(Z\right)>V\right\} & where\;Z=\left(X,Z\_\right) \end{array}$$

*Y*, is the outcome, in our case health, well‐being or loneliness, which takes the value of *Y*_1_ for those who engage in social participation and *Y*_0_ for those who do not, as shown in Equation (2). The outcomes are functions described by *μ*_*j*_ of observables (contained in *X*) and the error term (*U*_*j*_), where *j* = 1 indicates that the individual is “treated,” that is, socially participates. Equation (3) is the selection equation, which models selection into treatment via a latent index with 1 being an indicator function. Treatment is determined via *Z*, a combination of covariates contained in *X*, and instruments contained in *Z*_, which affect the probability of engaging in social participation, not the potential outcomes. In our case, these instruments are the availability of some community assets included as binary variables. Finally, within the selection equation in (3), the error term *V* is the unobservable resistance to treatment. We assume *V* has a continuous distribution which allows this equation to be written such that *P*(*Z*) > *U*_*D*_, where *P*(*Z*) is the propensity score, and *U*_*D*_ is the unobserved resistance to treatment.

One of the critical assumptions of the model is the conditional independence, implying that the following holds: $\left(U_{0},U_{1},V\right)⊥Z\_|X$. This infers that the errors are independent of the instrument given the covariates, which leads to the traditional IV assumptions of relevance, exclusion criteria, and monotonicity (Angrist & Imbens, 1995). We test the relevance and monotonicity assumptions statistically and argue for the exclusion restriction based on the theoretical concepts and literature described in the previous section. In addition to these arguments, we test the overidentifying restrictions formally.

Conditional independence is a sufficient condition on its own. However, it requires full support over the range of propensity scores defined in the closed interval [0,1]. We assume this will not be the case and that extreme propensity scores will generate a large amount of noise when estimating treatment effects. This is due to low densities of either treated or untreated observations (Carneiro et al., 2003). Therefore, we trim the scores by one percent at each tail to remove low densities from our common support region (Smith & Todd, 2005). We do this at both tails to retain the low and high propensities equally and protect the sample size and overall common support region.

Given this, an additional assumption of separability must hold, in which $E\left(U_{j}|V,X\right)=E\left(U_{j}|V\right)$. This implies that the errors relating to treated or untreated states are equal when conditional on both the unobserved resistance to treatment and covariates and conditional on just the unobserved resistance to treatment. This assumption has two implications: first, the treatment effects can be unconditionally estimated across the common support region on the covariates; second, the treatment effects can be separated into observable and unobservable components.

Given this, we can rewrite Equation (2) as Equation (4) to determine the treatment effects of social participation across propensity scores (*p*). We assume the functional form of the covariates within *μ*_*j*_(*X*) is linear, meaning that they have the following form, *xβ*_*j*_. The expected value of the outcome is evaluated at set values of the covariates and propensity scores. The second line shows that the estimation of the outcome has two components. The first component is the observable part which relates to the covariates, and the second is the unobservable part, the error terms. The unobservable components *U*_1_ − *U*_0_ are conditional on the unobserved resistance to treatment from the first stage (Andresen, 2018).

(4) $$\begin{array}{l} E\left\{Y|X=x,P\left(Z\right)=p\right\}=E\left\{Y_{0}+D\left(Y_{1}-Y_{0}\right)|X=x,P\left(Z\right)=p\right\}\\ =x\beta_{0}+x\left(\beta_{1}-\beta_{0}\right)p+pE\left(U_{1}-U_{0}|U_{D}\leq p\right) \end{array}$$

To estimate the marginal treatments effects, a derivative of Equation (4) is taken with respect to *p*, and evaluated at *u*. This is shown in Equation (5), where we have the returns to health, well‐being and loneliness of social participation at each quantile of unobserved resistance to treatment (*U*_*D*_ = *u*) conditional on *X* = *x*. Similarly, to Equation (4) there are the unobserved and observable components. The treatment effects are the average gains for individuals with values of covariates *X* = *x* at a certain unobserved resistance to treatment, *U*_*D*_ = *u*.

(5) $$MTE\;\left(x,u\right)=x\left(\beta_{1}-\beta_{0}\right)+E\left(U_{1}-U_{0}|U_{D}=u\right)$$

The coefficients in Equation (5) represent the observable, $x\left(\beta_{1}-\beta_{0}\right)$, and unobservable, $E\left(U_{1}-U_{0}|U_{D}=u_{d}\right)$, components. Together, they jointly indicate whether we have heterogenous treatment effects. We can test these forms of “observed” and “essential” heterogeneity separately.

Observed heterogeneity implies that the estimated effects of participation vary by the levels of covariates. Essential heterogeneity indicates differences in unobserved characteristics conditional on unobserved resistance to treatment between the two groups. The value of this coefficient indicates the selection effect of social participation relating to the outcomes. Positive values indicate that there is negative selection, meaning those with higher resistance, that is, lower probabilities of social participation, have higher returns. This selection effect determines the gradient of the marginal treatment effects curve when plotted, whilst the covariates' values determine the intercept's position.

### 3.3 Testing the model assumptions

We test the relevance assumption using Wald's chi‐squared test to assess if the instruments predict social participation (Gregory & Veall, 1985). In theory, the exclusion criteria are untestable as the outcomes are not jointly observed (Angrist et al., 1996), meaning $Y_{i}\left(D_{i}\right)=Y_{i}\left(Z,D\right)$. The choice of instruments should be based on conceptual arguments and previous literature, but can be supported with tests of overidentifying restrictions. We use Sargan's chi‐squared test (Sargan, 1958), which is based on the residuals from the two‐stage model and the cross‐product of the instruments. This is a necessary condition for the exclusion criteria, but it is not sufficient.

In addition, we can assess whether social participation is endogenous. We use the Wooldridge robust test score as this is appropriate with robust standard errors (Wooldridge, 1995). The test assesses the consistency of the estimated effect of social participation between the instrument variable model and OLS.

The monotonicity assumption implies that $\mathrm{Pr}\left(D=1|Z=z\right)\geq \mathrm{Pr}\left(D=1|Z=z^{\prime }\right)$, for all *z* > *z* ^′^. The process of testing this assumption is complicated by using multiple instruments (Mogstad & Torgovitsky, 2018). This is due to a non‐homogenous choice between instruments; one individual may be more likely to engage in social participation if a place of worship is nearby, whereas another if there is a community center nearby. Given this, we assess if social participation increases with the number of assets available. Instead of *Z*_ being a vector of binary variables of the different assets, we use a count of assets available with a range of [0,2]. We then re‐estimate the first‐stage model using the count as a factor variable. If the coefficients on the count of community assets are monotonically increasing, this supports the monotonicity condition for our instruments. This implies that the availability of more assets does not decrease the probability of engaging in social participation.

The marginal treatment effects models also require tests for both observed and essential heterogeneity. These tests are undertaken on the coefficient estimates from Equation (5). For observed heterogeneity, the test is an *F*‐test on the coefficients on the observables, with a null hypothesis of $H_{0}:x\left(\beta_{1}-\beta_{0}\right)=0$. If we can reject the null hypothesis, then this indicates that the effects of social participation vary by the covariates.

Essential heterogeneity relates to whether there are differences in unobserved characteristics conditional on unobserved resistance to treatment between the two groups. This is the Inverse Mills Ratio for the selection effect, and a *t*‐test is implemented on the coefficient of this term. We test the null hypothesis of $H_{0}:E\left(U_{1}-U_{0}|U_{D}=u\right)=0$. If we can reject the null, this indicates significant variation in the unobservables relating to the outcomes and the unobserved resistance to treatment.

### 3.4 Parameters of interest

The marginal treatment effect model can estimate a range of functional parameters. The first describes how the marginal treatment effects vary across (unobserved) resistance to treatment. The range is dictated by the area of common support for the propensity scores. The estimated treatment effect can be interpreted as the average gain for a particular level of unobserved resistance to treatment. Higher (unobserved) resistance to treatment represents those with lower probabilities of engaging in social participation.

Additionally, the marginal treatment effects can be aggregated over the unobserved resistance to treatment to generate average parameters. These include the average treatment effect (ATE), which is the treatment effect at the mean value of the propensity score; the ATE on treated (ATT), which is the treatment effect with higher weighting toward higher propensity scores; and the ATE on the untreated (ATU) with higher weighting on lower propensity scores (Heckman & Vytlacil, 2005). These are estimated via integrating the marginal treatment effects using weights over the common support region, with the ATT based on low values of *u*_*d*_ (individuals who are more likely to participate) and ATU based on high values of *u*_*d*_ (individuals who are less likely to participate). Finally, the local ATE is also reported, which is the treatment effect for compliers in a traditional instrumental variable model (Angrist & Imbens, 1995). This is estimated by a shift in the instrument from *Z*_ to *Z*_^′^, that is, community asset becomes available, resulting in an effect on social participation.

The model can also estimate marginal policy‐relevant treatment effects (MPRTE). These represent simulated average gains to individuals of marginal shifts in a policy that will lead individuals to engage in social participation. These are incremental changes in the propensity score or instruments that affect the selection Equation (3), with three different policy effects (Carneiro et al., 2011). These shifts are summarized in Table 4, with the marginal changes denoted by *α* and the incremental margin at which distance an individual will now engage in social participation represented by *ε*.

**TABLE 4:** Policy changes and distance to the margin for marginal policy relevant treatment effects.

MPRTE	Policy change	Distance to the margin
1st	*Z*_ = *Z*_ + *α*	$\left|\mu_{D}\left(Z\right)-V\right|<\varepsilon$
2nd	*P*(*Z*) = *P*(*Z*) + *α*	$\left|P\left(Z\right)-U_{D}\right|<\varepsilon$
3rd	*P*(*Z*) = *P*(*Z*)(1 + *α*)	$\left|\frac{P\left(Z\right)}{U_{D}}-1\right|<\varepsilon$

*Source*: Carneiro et al. (2010).

The first MPRTE is a marginal change in the instruments in the first stage. This can be interpreted as a policy that would increase asset provision for all individuals by a small increment. Those individuals close to the margin would shift into treatment, and the treatment effects are their health, well‐being, and loneliness benefits due to social participation. The second MPRTE is an absolute increase in the propensity scores by a small amount. An example of this type of policy could be a government announcement that all individuals can take an hour off work to engage in social participation, increasing the probability of social participation for all. It does not mean all individuals would engage, but it allows them to do so if they wish. The third MPRTE is a relative increase, which would increase all propensity scores by a small percentage. These disproportionally benefit individuals with higher propensity scores. A policy example is an introduction of funding for social participation groups for which communities could apply. Those in the community who are already likely to engage would be more likely to seek access to resources to increase their engagement. Those individuals unlikely to attend in the first place would be less likely to seek the additional resources.

### 3.5 Sensitivity analysis

We perform several sensitivity analyses. First, we use bootstrapped standard errors instead of robust ones. Bootstrapped standard errors are preferred for the marginal treatment effects model as they account for uncertainty in the propensity score estimates in the first stage (Andresen, 2018). However, bootstrapping is not consistent with the probability weights we use in the primary analysis. To assess the robustness of the standard errors and the results, we run the models without the survey weight adjustments and compare the findings on key parameters using bootstrapped and robust standard errors.

In a second sensitivity analysis, we assess how robust the estimated effects of social participation are when we remove participation in some groups from the definition of social participation. In the first case, we remove sport and exercise group attendance from the measure of social participation as these may contribute directly to an individual's health. In addition, this removes individuals who only participate in those groups; if an individual participates in other groups on top of sport and exercise, they remain defined as participants.

In a third sensitivity analysis, we remove children's education, youth activities, and trade unions from the definition of social participation. This is to assess the consistency of the results to removal of groups that are accessible only to sub‐groups in the population. For example, we remove trade unions (because participation is focused on the employed population) and children's education and activities (because participation is focused on parents).

In the next sensitivity analysis, we consider the instrument choice. We highlight in Section 3.1 the theoretical reasons why we believe our instruments are valid here. Part of Section 3.3 outlines the statistical tests on these instruments. However, we conduct a further sensitivity analysis of the community assets that have statistically significant and positive conditional effect on social participation in our sample. Secondary schools, shops and chemists have a negative significant effect on social participation (Table A2, column 2); we do not assess them as they are counterintuitive to our mechanism of social participation and reflect either densely populated areas or higher needs of services (chemists) that are linked the outcomes. We assess two instruments used in the main analysis (community centers and places of worship), plus sports center, youth club, parks and public transport links. We individually test the relevance of these assets using a Wald's chi‐squared test with a *p*‐value threshold of 0.01. This *p*‐value was chosen to avoid weak instruments. We use Sargan's overidentification test for combinations of these assets and then assess the effect of these instruments on the estimated ATE. We compare these coefficients and statistics to the main model.

We also conduct two further sensitivity analyses on restricting the sample. First, we exclude individuals who live in rural areas because they may be less likely to walk to the nearest facilities. We next exclude individuals who responded either “yes” to the question “do you have any physical or mental health conditions or illnesses lasting or expected to last for 12 months or more?” or “yes—a lot” to the question “does your condition or illness/do any of your conditions or illnesses reduce your ability to carry out day‐to‐day activities?” These individuals may have limitations that affect their walking speed and may therefore not report asset availability in a comparable way to other respondents because of their health.

## 4 RESULTS

### 4.1 Summary statistics

We remove 2354 observations (4.4%) from the total sample of responses (*N* = 53,404) due to missing values in either outcomes, instruments or covariates. We omitted a further 1044 observations to trim the propensity scores to the region of common support, leaving a sample of 50,006 individuals.

The summary statistics for community asset availability by social participation status are shown in Table 5. Those who participate have higher availability of assets compared to non‐participants. These differences are statistically significant across all but one of the 14 types of assets. The magnitude of the differences is largest for the assets we use as instruments.

**TABLE 5:** Summary statistics of community assets split by social participation status.

	Participants	Non‐participants	Difference	*p*‐value
Instruments				
Church/place of worship	0.859	0.781	0.078	<0.001
Community center/hall	0.673	0.577	0.097	<0.001
Covariates				
Youth club/center/facility	0.304	0.249	0.056	<0.001
Sports center/facility/club	0.534	0.457	0.077	<0.001
Pub	0.889	0.856	0.033	<0.001
Park	0.868	0.848	0.020	<0.001
Public transport links	0.923	0.895	0.028	<0.001
Post office	0.810	0.795	0.015	0.002
Secondary school	0.565	0.549	0.016	0.007
Library	0.616	0.579	0.037	<0.001
General/grocery shop	0.907	0.900	0.007	0.055
Primary school	0.872	0.836	0.036	<0.001
Health center/GP practice	0.758	0.730	0.027	<0.001
Chemist	0.791	0.792	−0.000	0.935
Observations	35,438	14,568		

*Note*: Data from the Community Life Survey 2013–2020. Estimated using population weights. Significance tested in regression models assessing control variable on social participation binary variable.

The summary statistics for the outcomes and covariates are contained in Table 6. There are significant differences in the outcomes between participants and non‐participants. Compared to the non‐participants, those who participate are generally older, female, more likely to be from a BAME group, of higher education level, single, have higher income, have children, are a homeowner, and living in a less deprived area, south of England and rural locations.

**TABLE 6:** Summary statistics of outcomes and covariates split by social participation status.

	Participants		Non‐participants		Difference	*p*‐value
	Mean	*N*/(SD)	Mean	*N*/(SD)		
ONS4						
Life satisfaction	7.148	(1.883)	6.662	(2.229)	0.488	<0.001
Worthwhile	7.416	(1.947)	6.836	(2.284)	0.582	<0.001
Happiness	7.159	(2.142)	6.687	(2.425)	0.474	<0.001
Anxiety	6.524	(2.790)	6.357	(2.888)	0.167	<0.001
Self‐ assessed health	3.990	(0.827)	3.762	(0.936)	0.228	<0.001
Loneliness	3.507	(1.133)	3.377	(1.218)	0.131	<0.001
Age (in years)						
16–19	0.058	2050	0.051	799	0.007	0.033
20–24	0.07	2482	0.098	1524	−0.028	<0.001
25–34	0.153	5386	0.203	3153	−0.050	<0.001
35–49 (base)	0.257	9075	0.221	3433	0.036	<0.001
50–64	0.229	8098	0.233	3621	−0.004	0.451
65–69	0.066	2331	0.057	886	0.009	<0.001
70–74	0.061	2167	0.048	750	0.013	<0.001
75 +	0.105	3705	0.089	1377	0.016	<0.001
Female	0.518	18,271	0.492	7651	0.025	<0.001
BAME	0.137	4825	0.129	1999	0.008	0.030
Highest qualification						
No qualifications (base)	0.051	1502	0.164	2202	−0.113	<0.001
GCSEs	0.267	7844	0.358	4798	−0.091	<0.001
A levels	0.236	6943	0.225	3025	0.010	0.053
Degree	0.446	13,133	0.253	3390	0.194	<0.001
Marital status						
Married (base)	0.287	10,112	0.366	5687	−0.079	<0.001
Single	0.546	19,275	0.440	6845	0.106	<0.001
Divorced/separated	0.094	3311	0.103	1600	−0.009	0.006
Widowed	0.049	1734	0.049	758	0.000	0.892
Missing	0.024	861	0.042	653	−0.018	<0.001
Log. of household income	9.404	(0.968)	9.173	(0.939)	0.231	<0.001
Number of adults	2.305	(0.990)	2.322	(1.022)	−0.016	0.216
Number of children	0.502	(0.859)	0.404	(0.789)	0.098	<0.001
Homeowner	0.702	24,759	0.601	8758	0.138	<0.001
IMD quintile						
1—Most deprived	0.158	5572	0.246	3827	−0.088	<0.001
2	0.187	6585	0.221	3441	−0.035	<0.001
3 (base)	0.204	7198	0.203	3153	0.001	0.821
4	0.218	7699	0.173	2690	0.045	<0.001
5—Least deprived	0.233	8239	0.156	2431	0.077	<0.001
Region						
North East	0.044	1564	0.057	878	−0.012	<0.001
North West	0.129	4538	0.136	2108	−0.007	0.093
Yorkshire and Humberside	0.094	3328	0.107	1657	−0.012	0.001
East Midlands	0.084	2964	0.091	1421	−0.007	0.029
West Midlands	0.102	3591	0.109	1694	−0.007	0.052
East of England	0.111	3919	0.113	1757	−0.002	0.575
London (base)	0.159	5596	0.149	2310	0.010	0.010
South East	0.171	6022	0.146	2268	0.025	<0.001
South West	0.107	3773	0.093	1449	0.014	<0.001
Rural	0.207	7301	0.154	2393	0.053	<0.001
Year						
2013 (base)	0.21	7415	0.136	2119	0.074	<0.001
2014	0.044	1569	0.035	550	0.009	<0.001
2015	0.044	1554	0.037	580	0.007	<0.001
2016	0.141	4962	0.129	2003	0.012	0.005
2017	0.14	4947	0.143	2225	−0.003	0.454
2018	0.142	4994	0.154	2393	−0.012	0.002
2019	0.145	5125	0.147	2278	−0.001	0.725
2020	0.134	4728	0.218	3394	−0.084	<0.001
Observations	35,438		14,568			

*Note*: Base indicates the base category for inclusion in models. Adjusted for population weights. Significance tested in regression models estimating control variable on social participation binary variable.

Abbreviations: BAME, Black, Asian or minority ethnicity; GCSE, General Certificate of Secondary Education.

### 4.2 Instrumental variable tests

The results of the tests of endogeneity (full tests score) and overidentifying restrictions (Sargan's chi‐square test) are shown in Table 7. Across all outcomes, the null hypothesis of exogeneity is rejected, which indicates that social participation is endogenous to the outcomes. This suggests that OLS estimates would be biased. We cannot reject the null for the overidentifying restrictions for all outcomes. This is necessary but not sufficient for excludability.

**TABLE 7:** Endogeneity and overidentifying restrictions for the outcomes.

	(1)	(2)	(3)	(4)	(5)	(6)
	Life satisfaction	Worthwhile	Happiness	Anxiety	Health	Lonely
Robust test score (endogeneity)	49.369	29.100	32.136	22.304	6.604	21.248
*p*‐value	<0.001	<0.001	<0.001	<0.001	<0.001	<0.001
Sargan's chi‐squared score (overidentifying restrictions)	1.221	0.832	3.104	0.021	2.510	3.580
*p*‐value	0.269	0.362	0.078	0.885	0.113	0.058
Observations	50,006	50,006	50,006	50,006	50,006	50,006

*Note*: Models adjusted using population weights and robust standard errors. Models include individual, household, area and asset controls, see Table A1. Robust test score has a chi‐squared distribution and degrees of freedom equal to a number of endogenous regressors (*k* = 1) (Wooldridge, 1995). Sargan's chi‐squared test has a chi‐squared distribution and degrees of freedom equal to the number of instruments minus the number of endogenous regressors (*Z*_ − *k* = 1) (Sargan, 1958).

Reported in Table 8 are the average marginal effects of the instruments (column 1) on social participation and the tests for monotonicity (column 2), with the relevance assumption assessed for both. The instruments have positive and statistically significant effects on the probability of social participation (column 1), with places of worship having the largest magnitude. The chi‐squared score indicates we can reject the null, indicating that the instruments are relevant.

**TABLE 8:** Average marginal effects for first stage results with the test for relevance and monotonicity.

	(1)	(2)
Binary assets		
Church/place of worship	0.063***	
	(0.048–0.077)	
Community center/hall	0.031***	
	(0.020–0.041)	
Count of assets (base = 0)		
1		0.067***
		(0.049–0.085)
2		0.099***
		(0.080–0.118)
Wald's chi‐squared score		
(relevance)	116.437	113.915
*p*‐value	<0.001	<0.001
Observations	50,006	50,006

*Note*: 95% confidence intervals based on robust standard errors in parentheses, *p < 0.05, **p < 0.01, ***p < 0.001, models adjusted using population weights. Models include individual, household, area and asset controls, see Table A1. Wald's Chi‐squared test has chi‐squared distribution and degrees of freedom equal to number of instruments (*Z*_ = 2) (Gregory & Veall, 1985).

Contained in column 2 are the assessments of the monotonicity assumption. The instruments meet this criterion. With additional community asset availability, there is a monotonically increasing probability of engaging in social participation, and this selection of instruments is relevant to social participation. Additionally shown in Table A2 (column 1) are the average marginal effects of the covariates used as a control variable in the model on social participation. Some of these community assets, such as youth centers, sports centers, parks and transport links, positively affect social participation. Others, like secondary schools, shops and chemists, have negative effects.

### 4.3 Common support

**FIGURE 1:**
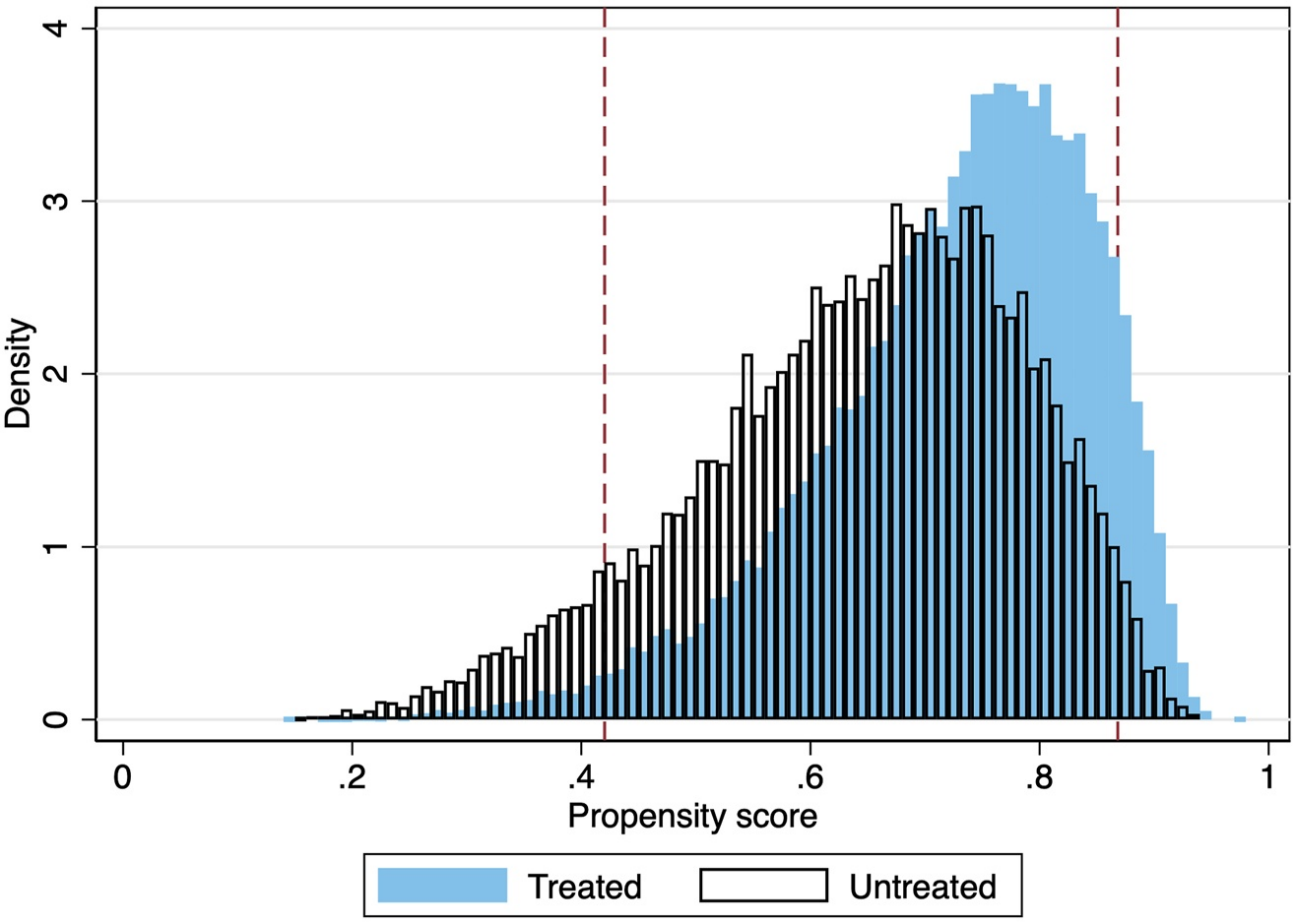
Propensity scores and common support region.

The distributions of propensity scores split by participation status are shown in Figure 1. The red dashed lines indicate where the propensity scores have been trimmed by one percent at each tail. Those individuals who engage in social participation have larger proportions of higher propensity scores, as expected. The trimming of the common support leaves the region of support in the closed interval [0.43, 0.86].

### 4.4 Marginal treatment effects tests and parameters

The estimated parameters and the tests for all the outcomes of the marginal treatment effects model are shown in Table 9. Across all the outcomes, the null hypothesis for observed heterogeneity can be rejected. For essential heterogeneity, the null can be rejected for life satisfaction, worthwhileness, happiness and health at the 5% significance level. For loneliness, we can reject the null at the 10% significance, with a *p*‐value of 0.075. However, we cannot reject the null for the anxiety outcome, with a *p*‐value of 0.49.

**TABLE 9:** Marginal treatment effects heterogeneity tests and treatment effect parameters.

	(1)	(2)	(3)	(4)	(5)	(6)
	Life satisfaction	Worthwhile	Happiness	Anxiety	Health	Lonely
ATE	2.173***	1.704***	2.032***	1.500**	0.403*	0.966***
	(0.429)	(0.442)	(0.479)	(0.576)	(0.175)	(0.231)
ATT	1.164	0.623	1.234	1.228	0.095	0.679*
	(0.635)	(0.641)	(0.705)	(0.822)	(0.254)	(0.324)
ATU	3.969***	3.631***	3.453***	1.966*	0.954***	1.466***
	(0.577)	(0.606)	(0.656)	(0.835)	(0.239)	(0.327)
LATE	3.015***	2.647***	2.697***	1.695**	0.665***	1.054***
	(0.404)	(0.427)	(0.458)	(0.580)	(0.169)	(0.232)
1st MPRTE	0.321***	0.293***	0.279***	0.163*	0.077***	0.120***
	(0.048)	(0.050)	(0.054)	(0.069)	(0.020)	(0.027)
2nd MPRTE	0.342***	0.311***	0.297***	0.175*	0.081***	0.130***
	(0.051)	(0.054)	(0.058)	(0.074)	(0.021)	(0.029)
3rd MPRTE	0.391***	0.358***	0.338***	0.198*	0.093***	0.147***
	(0.060)	(0.063)	(0.068)	(0.086)	(0.025)	(0.034)
Inverse Mills ratio	5.286**	5.598**	4.176*	1.612	1.572*	1.605
	(1.732)	(1.754)	(1.942)	(2.336)	(0.693)	(0.900)
*p*‐value						
Observed heterogeneity	<0.001	<0.001	<0.001	<0.001	<0.001	<0.001
Essential heterogeneity	0.002	0.001	0.032	0.490	0.023	0.075
Observations	50,006	50,006	50,006	50,006	50,006	50,006

*Note*: Robust standard errors in parentheses, *p < 0.05, **p < 0.01, ***p < 0.001, models adjusted using population weights. Models include individual, household, area and asset controls, see Table A1. 1st indicates an incremental shift in the instrument, 2nd indicates an incremental shift in the absolute value of the propensity scores, and 3rd indicates an incremental shift in the relative value of the propensity scores (see Table 4).

Abbreviations: ATE, average treatment effect; ATT, average treatment effect on treated; ATU, average treatment effect on untreated; LATE, local average treatment effect; MPRTE, marginal‐policy relevant treatment effect.

The positive coefficients on the Inverse Mills Ratio coefficient across all outcomes indicate negative selection. This means that those with high resistance to treatment, that is, low probabilities of participating in social participation, have greater returns to their outcomes. This is further supported by the ATE on untreated (ATU) having higher returns than the ATE on treated (ATT).

Across the ONS4 measures of well‐being, the highest return to social participation for the average individual is for the happiness score (column 3), with a 2.03 increase compared to non‐participants. This increase is similar to that for life satisfaction. The estimated returns are the lowest for anxiety. The ATE for self‐assessed health (column 5) is 0.40, with estimated effects on the treated of 0.1 and on the untreated of 0.95. This indicates disproportionate health benefits for those less likely to engage in social participation. A similar pattern is exhibited for the loneliness measure as engaging in social participation means individuals report feeling lonely less frequently.

The MPRTE show that incremental shifts in the first stage model have significant returns to individuals with narrow margins between their propensity score and unobserved resistance to treatment. For a universal increase in community asset provision (1st MPRTE), there is an additional increase of 0.32 in life satisfaction score across individuals and a 0.08 increase in self‐assessed health. For an absolute increase in propensity scores (2nd MPRTE), there is an 0.34 increase in life satisfaction score across all individuals and a 0.08 increase for health. A relative increase in propensity scores (3rd MPRTE), which disproportionately affects the high propensity scores, shows the greatest return across all outcomes. This effect shows a 0.39 increase in life satisfaction across all individuals and a 0.09 increase in health score. These treatment effects indicate a small shift in the instrument provision, and propensity scores have significant returns to the individuals that may be affected by different policies.

Table A3 in the appendix contains the coefficients on all covariates in the models. We find different treatment effects for outcomes across observable covariates. For example, across all outcomes, we find higher returns to social participation for those on a low income and those who live alone or with no children. For life satisfaction, worthwhileness and health, the results suggest that individuals with no education have significantly more to gain from social participation than those with educational attainment of either General Certificate of Secondary Education, A Levels or Degree level. Additionally, for those outcomes we find that individuals who do not own their home have higher returns for social participation than homeowners.

### 4.5 Marginal treatment effect plots

**FIGURE 2:**
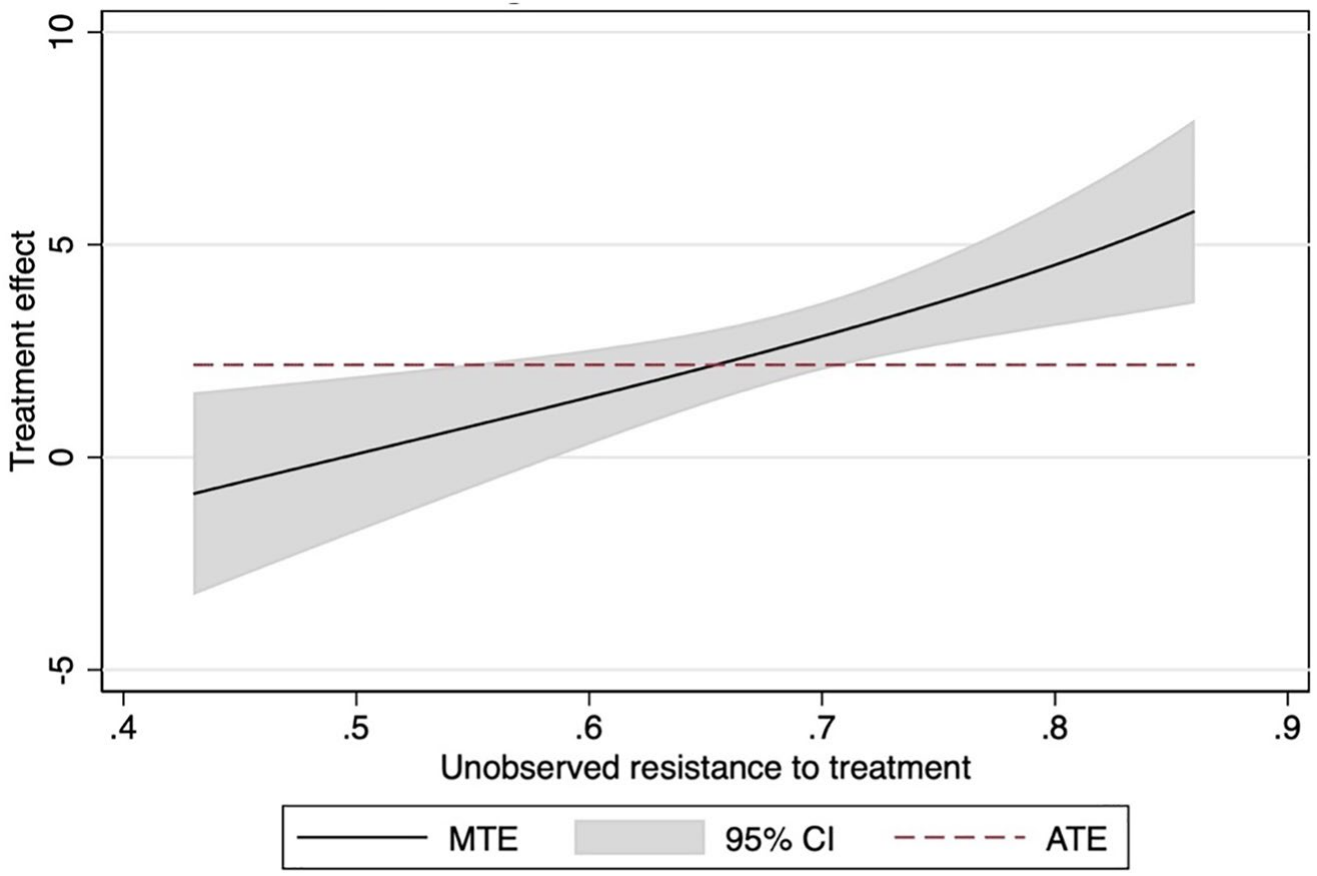
Marginal treatment effects curve for life satisfaction score. 95% confidence intervals based on robust standard errors.

The plot of marginal treatment effects for the life satisfaction component of the ONS4 is provided in Figure 2. The marginal treatment effect curves for the other three components of the ONS4 are contained in the appendix in Figures A1–A3. At the lower end of unobserved resistance to treatment, individuals have insignificant effects of social participation on life satisfaction, with *U*_*d*_ < 0.59, representing the individuals with the highest propensity scores. As unobserved resistance to treatment increases, so does the magnitude of the treatment effect. For example, those with unobserved resistance to treatment >0.8, representing individuals whose estimated probability of engaging in social participation is 20% or less, have treatment effects of over 4.5 points.

**FIGURE 3:**
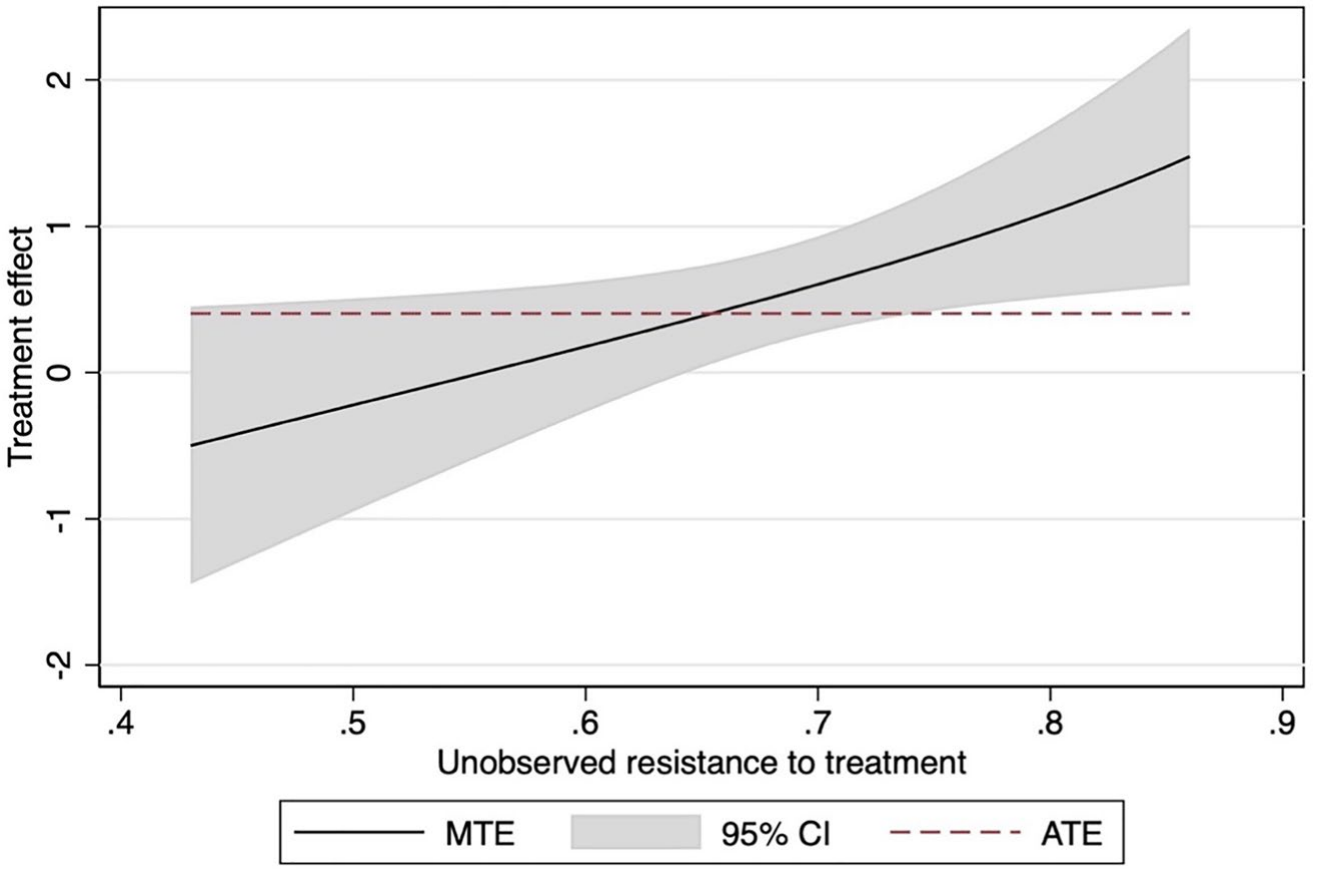
Marginal treatment effects curve for self‐assessed health. 95% confidence intervals based on robust standard errors.

Contained in Figure 3 is the marginal treatment effects plot for self‐assessed health. There are significant treatment effects for values of unobserved resistance to treatment of 0.65 and over. The upward slope indicates this negative selection effect, with high unobserved resistance to treatment reaping the highest returns to social participation. A similar pattern is shown for the loneliness outcome in Figure A4 in the appendix, with treatment effects significant with *U*_*d*_ ≥ 0.57.

### 4.6 Sensitivity analysis

In an unweighted analysis in Table A4, we can compare bootstrapped standard errors to robust standard errors. The bootstrapped standard errors are larger across all the outcomes. However, the differences are minor in magnitude, and all parameters remain significant.

Contained Table A5 is the first stage results showing that our instruments are still relevant (column 1), and monotonicity holds (column 2) when we removed the sports and exercise attendance from our measure of social participation. Table A2 (column 2) shows the effects of all the assets on the social participation measure; the instruments stayed consistent with the results using the broad measure of participation (column 1). As expected, the removal of sports and exercise groups decreased the effect of having a sports center nearby. We further show in Table A6 that social participation is still endogenous to all outcomes and the instruments remain valid. We also present the area of common support in Figure A5, which shows a leftward shift in the distribution of propensity scores and a lower peak of treated densities from Figure 1. Additionally, the range of propensity scores is larger, particularly at lower propensity scores.

Table A7 contains the parameters from the marginal treatment effects model and tests for observed and essential heterogeneity. We cannot reject the null for happiness, anxiety and loneliness for essential heterogeneity. Nevertheless, the parameters show that the models are still broadly robust, with similar magnitudes and significance levels. Removing sports and exercise attendance from our social participation measure leads to a reduction in the health returns regarding the ATE on untreated and the local ATEs.

We also examined the effects of removing participation in trade unions and children's education and activities from the measure of social participation. As before, Table A5 contains the first stage results and shows that our instruments are still relevant (column 3), and monotonicity holds (column 4). Table A2 (column 3) contains results for the assets on the social participation measure. The instruments stay consistent with the broad measure (column 1). Including only those groups accessible to all increases the effect of a sports center nearby and decreases the impact of youth centers slightly. Table A6 shows that social participation is still endogenous to all outcomes due to significant values from the Robust test score. The instruments are valid as we cannot reject the null for overidentifying restrictions. Figure A6 shows the common support area, which shows similar densities to that of Figure 1. Table A8 has the parameters and model tests for the marginal treatment effects model. These results show marginally higher returns, as evidenced by larger parameters and coefficients on the Inverse Mills Ratio. This could be because the type of groups contributes less to outcomes, or individuals have some secondary effects from either being employed or having children. Nonetheless, all the parameters remain the same significance, with only small changes in estimated parameters.

Table A9 contains the relevance testing on community assets that had positive and statistically significant effects on social participation within A2, column 1. For public transport links and parks, the chi‐squared score does not meet the threshold of a *p*‐value of 0.01. Table A10 shows the instrument choice we used for the analysis are the only combinations that comply with the necessary conditions for excludability. We show the effect on the ATEs that they are qualitatively the same in Table A11. However, the estimated values are larger compared to the main model.

In the next set of sensitivity analyses, we restrict the sample size to population groups we hypothesize would have different access to community assets. Table A12 contains the marginal treatment effects model restricting the sample to urban dwellers only (41,171 respondents, 82% of the sample). We show that our results are robust to the main models in terms of sign and significance.

We next restrict the sample to 45,751 respondents (91% of the sample) with no limiting long term conditions. The first‐stage results are reported in Table A13. These show that the instruments still satisfy both the relevance and monotonicity assumptions. The chi‐squared test of instrument relevance and the estimated effects of the instruments on social participation are larger than in the main model (Table 8), indicating a better model fit for those with better mobility. The results of the marginal treatment effects models for this sub‐sample are contained in Table A14. The results are similar to the main models in terms of sign and significance, but the magnitude of the effects are smaller, likely indicating the lower variability of health status in this sub‐sample.

## 5 DISCUSSION

We investigated the returns of social participation to health, well‐being and loneliness for adults in England using a nationally representative sample of the general population. We show that community asset infrastructure is an appropriate instrument for social participation across all the outcomes in terms of relevance, monotonicity, validity and endogeneity. We found that social participation was linked to improved health and well‐being and reduced loneliness. These effects were heterogenous for health, life satisfaction, feeling worthwhile and happiness with negative selection effects. This indicates that those who are less likely to engage have higher returns to their outcomes compared to those with higher propensity scores. Incremental shifts in the first stage through the MPRTE indicate positive returns to outcomes, particularly for relative changes in the propensity scores.

### 5.1 Relationship with past findings

This paper supports past research into social participation. The first stage results support the notion of social infrastructure acting as either a facilitator of social participation (Roskruge et al., 2012), with the survey results validating the two key components to access: proximity and individuals can access via walking (Sáinz‐Ruiz et al., 2021). We show that places of worship and community centers within a 15–20‐min walk positively impact the probability of social participation. These were assets identified in previous findings using the Community Life Survey assessing predictors of social participation (Wilding et al., 2022). Our sample uses an additional two waves of the Community Life Survey, and we find two other predictors of social participation, parks and public transport links. We excluded them from the set of instruments as we argued they were likely to violate the necessary exclusion criteria.

This research supports previous studies and adds additional evidence. For example, the findings support that from a survey of older people in Salford, which found participation improved quality of life (Munford, Panagioti, et al., 2020; Munford, Wilding, et al., 2020), and similar results found in a study of the elderly population in England (Steffens et al., 2016). Additionally, we found that those participants had reduced loneliness compared to non‐participants.

We find similar evidence of self‐selection as identified by Dawson‐Townsend, 2019, in Switzerland. This is particularly evident in Table 5, where we show the summary statistics stratified by participation status. This indicates that those who participate have higher educational levels and income, further reinforcing this self‐selection issue, and those who participate have different underlying characteristics than non‐participants. Even when controlling for this within the models, this further motivates the use of the instrumental model. This type of modeling is further supported by the endogeneity of social participation identified in Table 7, highlighted as drawbacks of past findings (Ichida et al., 2013; Tomioka et al., 2017).

### 5.2 Strengths

This research has several strengths coming from the data and methodology. The first arises from the survey asking about community asset infrastructure. This feature is not available in any other surveys. This leads to a second strength; the use of this specification to overcome endogeneity around social participation. This allows for interpretations beyond just associations between outcomes and social participation.

Using survey weights to adjust the results to England's population characteristics gave the findings external validity. These are used in all models to adjust the sample to England's population and to assess the effect of social participation in a general population.

The methods used in this research allow for heterogenous treatment effects; this feature is important for assessing who can benefit most from social participation. However, this has yet to be undertaken in other studies and has demonstrated the need to consider carefully whom to direct social participation to aid in reducing inequalities.

### 5.3 Limitations

Several limitations of this study arise from the data and survey. The first is measurement error in our instrumental variable as it is self‐reported. This is multifaceted and can occur in different ways. Firstly, individuals who use community assets for social participation activities are more likely to be able to accurately judge if they are within a 15–20 min walking distance. This could mean that we are not correctly capturing information on asset infrastructure availability for those who do not report that they participate. If this is the case, there may be differential measurement error present in the data. A priori, we cannot hypothesize how this may affect our estimated effect sizes. One way to assess this would be to look at individuals who respond “don't know” when asked about community asset availability within the survey. This could be linked to not engaging in social participation. However, only 16 individuals out of 56,944 provided this response to the survey question.

Secondly, individuals were asked whether the assets are within a 15–20‐min walk radius, which will vary from individual‐to‐individual. For example, this walk time could be correlated with the health of individuals, as those with less mobility due to long‐term health conditions may have fewer assets in their 15–20 min walk‐time if they walk more slowly. To address this possible concern, we control for age in our models, which is correlated with several long‐term conditions. However, we do not directly include a variable to indicate whether an individual has a long‐term health condition as this is likely to be associated with our outcomes. In additional analyses, we excluded people who reported that they had a long‐term health condition that limits their day‐to‐day activities and we obtain very similar results to the main models. We argue here that the fact that the results for the whole sample and the sample who are not limited in their mobility are very similar may imply that the correlation between mobility, availability of assets, and health may not be a major source of concern.

The final reason for possible bias in our instrument is related to “back‐door” contamination through the reporting of the asset availability. There is a potential that individuals do not report community assets being available as justification for not engaging in social participation. This risk is less likely when the survey questions relating to availability of assets are asked before the questions about their own social participation. As the data we use are collected via an online questionnaire, there is a possibility that respondents go back and edit their responses to earlier questions to justify their responses to later questions, but we believe this is unlikely.

Further limitations come from the geographical identifier available in the dataset. The current identifier is Government Office Region, which divides England into nine areas. Due to this, we cannot include other area‐level variables from external sources. For example, secondary data is available for funding community assets and local services at the local authority level, which would enrich the estimation.

The loneliness outcome has issues with reporting. First, there is stigma for individuals reporting their loneliness, and this survey contains a direct measure. This means individuals could be underreporting their loneliness, and indirect measures are better at measuring as they do not include the word “lonely” (Shiovitz‐Ezra & Ayalon, 2012).

The coefficients at the extreme ends of our common support and for the average treated on untreated were large. Particularly across the ONS4 measures, this could indicate that our results have issues from noise generated by low densities of propensity scores at the edges of the common support region.

Further limitations from the dataset relate to it being a repeated cross‐section rather than longitudinal. Ideally, we would like to have followed individuals over some years to assess the role of social participation in the outcomes studied. Unfortunately, we could not find panel data on community asset infrastructure.

### 5.4 Implications

The findings show the benefits of social participation in a general population and suggest that future policies around social participation should focus on individuals who are less likely to participate. It has been proposed that engagement in groups that use these assets could reduce health and well‐being inequalities (Foot & Hopkins, 2010). This is demonstrated here, as encouragement of participation for those with low propensity scores would lead to higher marginal returns and improvements in their outcomes that would reduce health inequalities.

It is important to note, however, that preferences around social participation will vary from individuals with a low to a high probability of engaging. There is a trade‐off between the benefits of social participation and the forgone utility from other uses of that time. We cannot assess potential disutility from switching time into social participation, which may require less time at work or at leisure. We have implicitly adopted an extra‐welfarist perspective (Brouwer et al., 2008), as we have measured benefits only in terms of health and wider well‐being outcomes.

One means of encouraging this participation is through asset infrastructure, as this research has shown that this is an essential predictor of participation. However, this is two‐fold, as it requires having these community assets nearby and awareness of social participation. This awareness is better coming from the local communities than a centralized approach (McKnight & Kretzmann, 1993). This is because they better understand their area and the needs of the individuals.

These effects are also evident in the marginal policy treatment effects. For example, increasing access for those less likely to participate can enable higher participation rates and, in turn, improve outcomes. Other policies that increase the probability of attending by making it easier for individuals to attend or encouraging communities to engage also have the scope to produce benefits. However, if the objective is to reduce inequalities, they need to benefit those less likely to participate rather than those who need a small encouragement to attend.

## CONFLICT OF INTEREST STATEMENT

The authors declare no conflicts of interest.

## Supporting information

Supporting Information S1

## Data Availability

The data that supports the findings of this study are openly available in UK data service at: 2013/14 http://doi.org/10.5255/UKDA‐SN‐7737‐1, reference number 7737. 2014/15 http://doi.org/10.5255/UKDA‐SN‐7900‐1, reference number 7900. 2015/16 http://doi.org/10.5255/UKDA‐SN‐8124‐1, reference number 8124. 2016/17 http://doi.org/10.5255/UKDA‐SN‐8294‐1, reference number 8294. 2017/18 http://doi.org/10.5255/UKDA‐SN‐8478‐1, reference number 8478. 2018/19 http://doi.org/10.5255/UKDA‐SN‐8584‐1, reference number 8584. 2019/20 http://doi.org/10.5255/UKDA‐SN‐8767‐1, reference number 8767. 2020/21 http://doi.org/10.5255/UKDA‐SN‐8867‐1, reference number 8867.

## ENDNOTE

^1^
Household income was approximated using an interval regression from categorical bands from the Community Life Survey in order to get a continuous measure to equivalise and deflate. Full details are available on request.
