# Supplementary material for: Estimating the heterogeneous health and well‐being returns to social participation
Source: Health Econ. 2023 May 5;32(9):1921–40. doi: 10.1002/hec.4695 (PMC10946765; doi:10.1002/hec.4695)
Supplement: Supplementary file 1 — Supporting Information S1 [file HEC-32-1921-s001.docx]

# Appendix

Figure A 1: Marginal treatment effects curve for worthwhile score

**
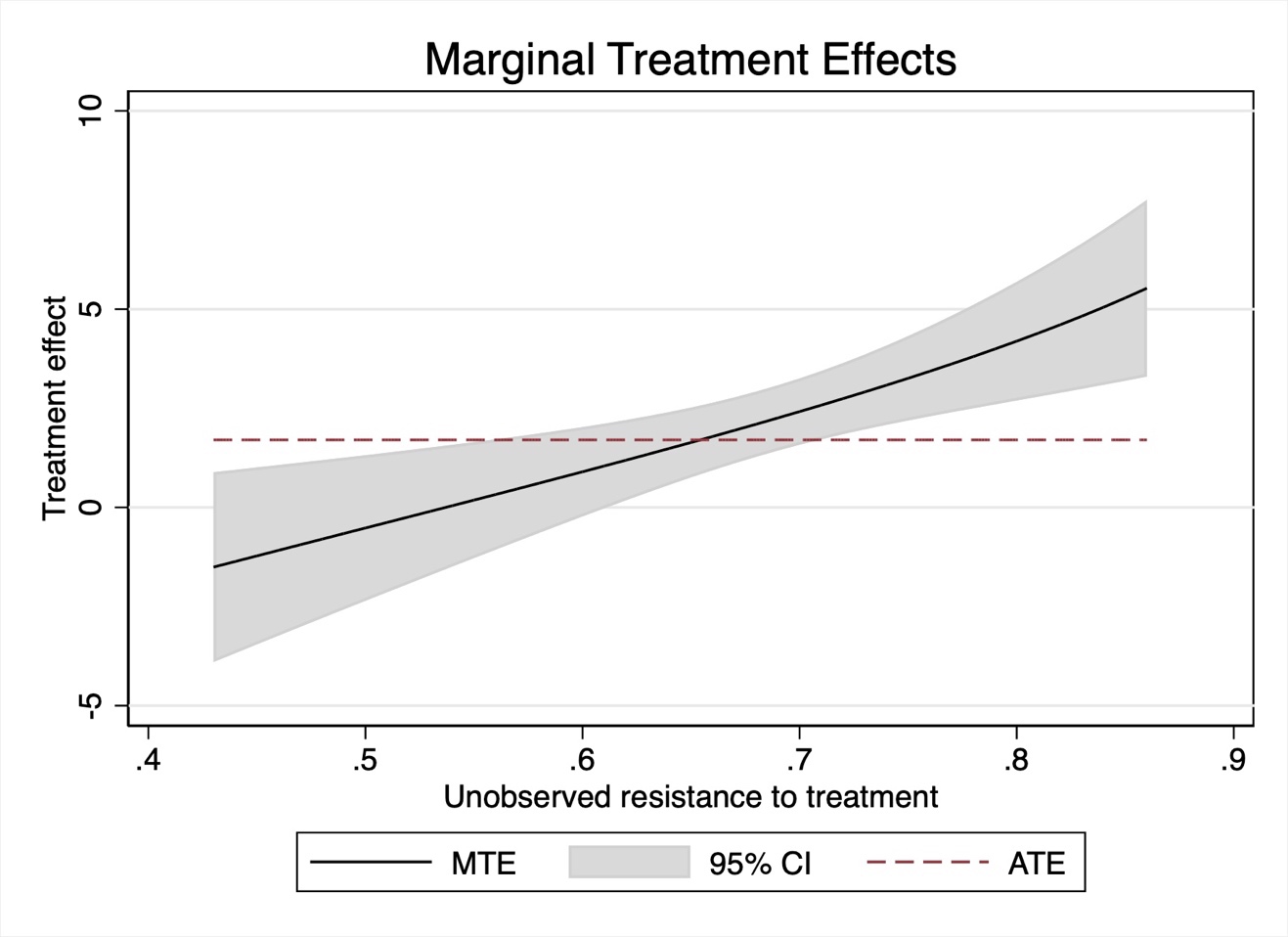
**

**Note:** 95% Confidence Intervals based on robust standard errors

Figure A 2: Marginal treatment effects curve for happiness score


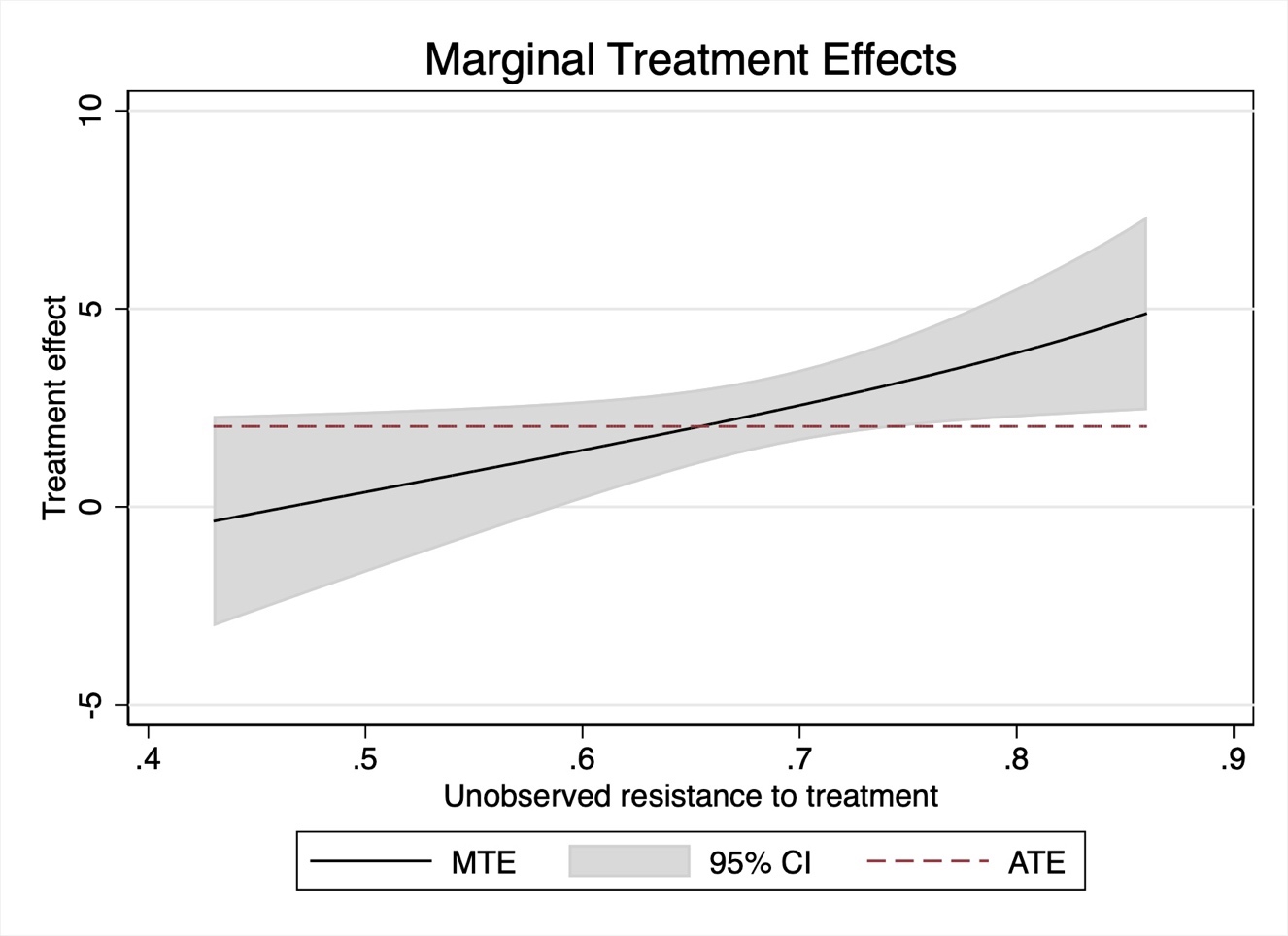


**Note:** 95% Confidence Intervals based on robust standard errors

Figure A 3: Marginal treatment effects curve for anxiety score


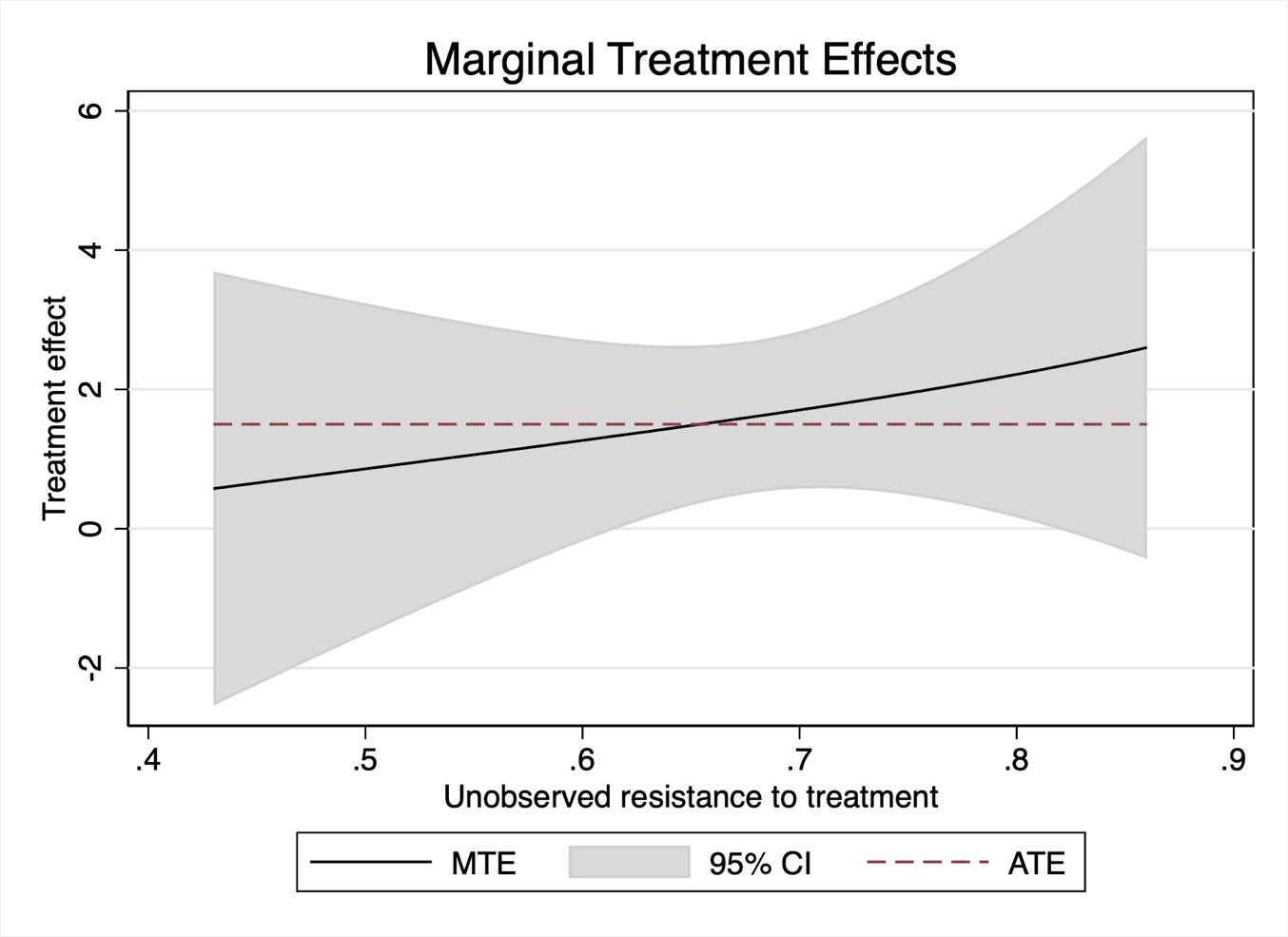


**Note:** 95% Confidence Intervals based on robust standard errors

Figure A 4: Marginal treatment effects curve for loneliness score


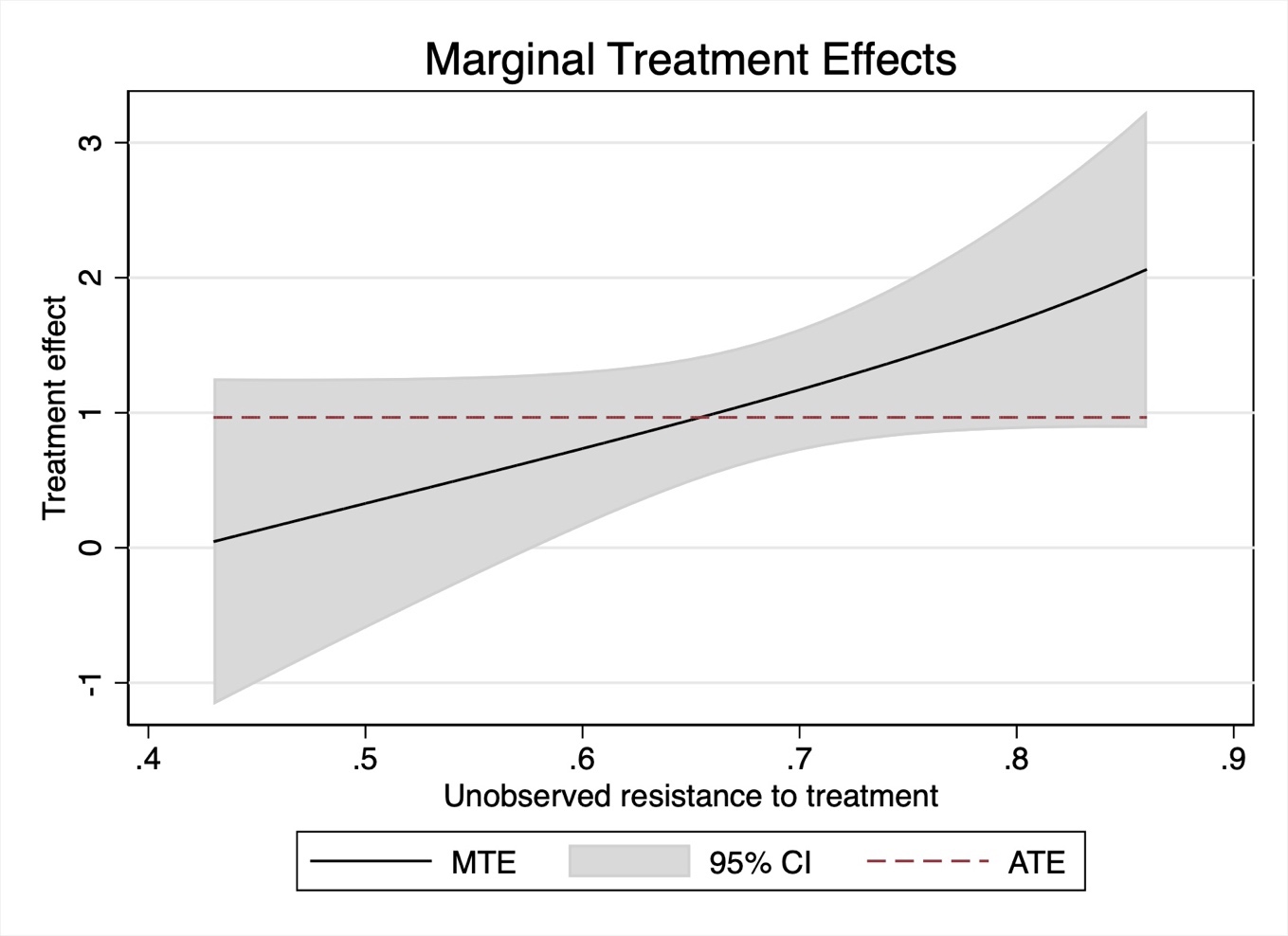


**Note:** 95% Confidence Intervals based on robust standard error

Figure A 5: Common support graph for social participation measure without sports and exercise groups' attendance

Figure A 6: Common support graph for social participation measure without trade union, children’s groups and youth activities attendance

Table A 1: Individual, Household and Area Characteristics

| **Characteristics** | **Detail** | **Variable Type** |
| --- | --- | --- |
| **Age** | Categories: | Categorical |
|  | 16 to 19 years |  |
|  | 20 to 24 years |  |
|  | 25 to 34 years |  |
|  | 35 to 49 years (Base Category) |  |
|  | 50 to 64 years |  |
|  | 65 to 69 years |  |
|  | 70 to 74 years |  |
|  | 75 years + |  |
| **Gender** | Female =1 | Binary |
| **Ethnicity** | Black and Asian Minority Ethnicity (BAME) =1 | Binary |
| **Marital Status** | Categories: | Categorical |
|  | Single |  |
|  | Married (Base Category) |  |
|  | Divorced/Separated |  |
|  | Widowed |  |
|  | Not Reported |  |
| **Highest Qualification Obtained** | Categories: | Categorical |
|  | No Qualifications (Base Category) |  |
|  | GCSEs |  |
|  | A Levels |  |
|  | Degree |  |
| **Household Income** | Logarithmic Value | Continuous |
| **House Composition** | Number of Adults | Count |
|  | Number of Children | Count |
| **Housing Tenue** | Homeowner=1 | Binary |
| **Index of Multiple Deprivation** | Categories: | Categorical |
| **Quintile** | 1 - Most |  |
|  | 2 |  |
|  | 3 (Base Category) |  |
|  | 4 |  |
|  | 5 - Least |  |
| **Region** | Categories: | Categorical |
|  | North East |  |
|  | North West |  |
|  | Yorkshire and Humberside |  |
|  | East Midlands |  |
|  | West Midlands |  |
|  | East of England |  |
|  | London |  |
|  | South East |  |
|  | South West |  |
| **Rurality** | Rural=1 | Binary |
| **Survey Year** | Categories: | Categorical |
|  | 2013 (Base Category) |  |
|  | 2014 |  |
|  | 2015 |  |
|  | 2016 |  |
|  | 2017 |  |
|  | 2018 |  |
|  | 2019 |  |
|  | 2020 |  |

Table A 2: Average Marginal Effects from first stage results of community assets on social participation

|  | (1) | (2) | (3) |
| --- | --- | --- | --- |
|  | All Groups | No Sport and Exercise Groups | No Trade Unions or Children's Education or Activities |
| **Instruments** |  |  |  |
| Church/place of worship | 0.063*** | 0.067*** | 0.067*** |
|  | (0.048 to 0.077) | (0.052 to 0.082) | (0.052 to 0.082) |
| Community centre/hall | 0.031*** | 0.035*** | 0.032*** |
|  | (0.020 to 0.041) | (0.024 to 0.047) | (0.021 to 0.043) |
| **Covariates** |  |  |  |
| Sports centre/facility/club | 0.041*** | 0.019** | 0.048*** |
|  | (0.031 to 0.052) | (0.007 to 0.030) | (0.038 to 0.059) |
| Youth club/centre/facility | 0.021*** | 0.032*** | 0.020** |
|  | (0.009 to 0.033) | (0.020 to 0.045) | (0.008 to 0.033) |
| Pub | -0.004 | -0.020* | -0.002 |
|  | (-0.020 to 0.011) | (-0.037 to -0.004) | (-0.018 to 0.014) |
| Park | 0.016* | 0.020* | 0.018* |
|  | (0.001 to 0.031) | (0.004 to 0.036) | (0.002 to 0.034) |
| Public transport links | 0.019* | 0.006 | 0.021* |
|  | (0.000 to 0.038) | (-0.014 to 0.026) | (0.001 to 0.040) |
| Post Office | -0.005 | -0.001 | -0.011 |
|  | (-0.019 to 0.009) | (-0.016 to 0.014) | (-0.026 to 0.003) |
| Secondary school | -0.014** | -0.017** | -0.018** |
|  | (-0.025 to -0.003) | (-0.028 to -0.006) | (-0.029 to -0.007) |
| Library | 0.003 | 0.018** | 0.000 |
|  | (-0.009 to 0.014) | (0.006 to 0.030) | (-0.011 to 0.012) |
| General/grocery shop | -0.019* | -0.023* | -0.027** |
|  | (-0.037 to -0.000) | (-0.043 to -0.004) | (-0.045 to -0.008) |
| Primary school | 0.005 | 0.002 | -0.000 |
|  | (-0.011 to 0.021) | (-0.015 to 0.019) | (-0.017 to 0.016) |
| Health centre/GP practice | 0.002 | -0.003 | 0.002 |
|  | (-0.012 to 0.016) | (-0.018 to 0.012) | (-0.013 to 0.017) |
| Chemist | -0.035*** | -0.036*** | -0.034*** |
|  | (-0.052 to -0.019) | (-0.053 to -0.019) | (-0.050 to -0.017) |
| Observations | 50,006 | 50,041 | 49,975 |

**Note:** 95% Confidence Intervals based on robust standard errors in parentheses, * p<0.05, ** p<0.01, *** p<0.001, models adjusted using population weights. Models include individual, household, area and asset controls, see table A 1. Number of observations in column 2 and 2 vary to column 1 due to trimming of common support region for social participation measure.

Table A 3: Covariates from marginal treatment effects model

|  | (1) | | (2) | | (3) | | (4) | | (5) | | (6) |  |
| --- | --- | --- | --- | --- | --- | --- | --- | --- | --- | --- | --- | --- |
|  | Life Satisfaction | | Worthwhile | | Happiness | | Anxiety | | Health | | Lonely | |
|  | $\beta_{0}$ | $\beta_{1}-\beta_{0}$ | $\beta_{0}$ | $\beta_{1}-\beta_{0}$ | $\beta_{0}$ | $\beta_{1}-\beta_{0}$ | $\beta_{0}$ | $\beta_{1}-\beta_{0}$ | $\beta_{0}$ | $\beta_{1}-\beta_{0}$ | $\beta_{0}$ | $\beta_{1}-\beta_{0}$ |
| Youth club | 0.168 | -0.187 | 0.167 | -0.179 | 0.162 | -0.148 | 0.064 | -0.066 | 0.127* | -0.106 | 0.029 | -0.045 |
|  | (0.145) | (0.198) | (0.149) | (0.203) | (0.161) | (0.220) | (0.194) | (0.270) | (0.058) | (0.079) | (0.077) | (0.106) |
| Sports centre | 0.172 | -0.108 | 0.261 | -0.239 | 0.137 | -0.037 | 0.024 | 0.069 | -0.088 | 0.124 | -0.032 | 0.089 |
|  | (0.156) | (0.204) | (0.160) | (0.209) | (0.176) | (0.231) | (0.207) | (0.276) | (0.063) | (0.083) | (0.081) | (0.107) |
| Pub | 0.044 | -0.017 | -0.017 | 0.031 | -0.050 | 0.025 | -0.123 | 0.284 | 0.040 | 0.011 | -0.027 | 0.088 |
|  | (0.171) | (0.242) | (0.170) | (0.242) | (0.185) | (0.262) | (0.207) | (0.301) | (0.068) | (0.097) | (0.088) | (0.127) |
| Park | 0.090 | 0.049 | -0.129 | 0.334 | -0.059 | 0.275 | 0.187 | -0.102 | 0.075 | -0.045 | -0.016 | 0.006 |
|  | (0.170) | (0.236) | (0.171) | (0.238) | (0.185) | (0.257) | (0.213) | (0.303) | (0.071) | (0.099) | (0.087) | (0.122) |
| Public transport links | -0.055 | 0.017 | 0.066 | -0.177 | -0.050 | 0.051 | 0.102 | -0.008 | 0.009 | 0.014 | 0.002 | -0.034 |
|  | (0.201) | (0.287) | (0.195) | (0.281) | (0.221) | (0.316) | (0.247) | (0.361) | (0.080) | (0.116) | (0.102) | (0.149) |
| Post Office | 0.072 | 0.019 | 0.259 | -0.171 | -0.006 | 0.096 | 0.052 | -0.103 | 0.074 | -0.046 | -0.025 | 0.089 |
|  | (0.149) | (0.205) | (0.152) | (0.210) | (0.165) | (0.228) | (0.192) | (0.271) | (0.062) | (0.086) | (0.079) | (0.111) |
| Secondary school | 0.200 | -0.163 | 0.230 | -0.162 | 0.161 | -0.120 | 0.521** | -0.611** | 0.071 | -0.062 | 0.085 | -0.038 |
|  | (0.122) | (0.165) | (0.126) | (0.171) | (0.137) | (0.187) | (0.161) | (0.223) | (0.050) | (0.068) | (0.064) | (0.088) |
| Library | 0.071 | -0.122 | 0.104 | -0.197 | 0.011 | 0.005 | -0.179 | 0.275 | 0.075 | -0.076 | -0.002 | -0.011 |
|  | (0.131) | (0.181) | (0.131) | (0.181) | (0.140) | (0.194) | (0.167) | (0.236) | (0.053) | (0.074) | (0.067) | (0.095) |
| General/grocery shop | 0.122 | -0.205 | 0.160 | -0.284 | 0.089 | -0.174 | 0.199 | -0.253 | 0.014 | -0.038 | 0.030 | -0.085 |
|  | (0.220) | (0.302) | (0.210) | (0.289) | (0.234) | (0.323) | (0.260) | (0.366) | (0.083) | (0.115) | (0.104) | (0.146) |
| Primary school | 0.007 | -0.143 | 0.109 | -0.211 | 0.072 | -0.170 | 0.203 | -0.306 | -0.036 | 0.015 | -0.123 | 0.136 |
|  | (0.172) | (0.244) | (0.171) | (0.243) | (0.186) | (0.266) | (0.218) | (0.316) | (0.070) | (0.100) | (0.089) | (0.128) |
| Health centre/GP practice | 0.048 | -0.036 | -0.023 | 0.038 | 0.090 | -0.144 | -0.231 | 0.230 | -0.043 | 0.032 | 0.030 | -0.028 |
|  | (0.147) | (0.205) | (0.153) | (0.214) | (0.164) | (0.231) | (0.196) | (0.280) | (0.061) | (0.086) | (0.079) | (0.112) |
| Chemist | -0.241 | 0.400 | -0.400* | 0.612* | -0.236 | 0.366 | -0.114 | 0.266 | -0.211** | 0.241* | -0.057 | 0.075 |
|  | (0.174) | (0.243) | (0.178) | (0.249) | (0.194) | (0.271) | (0.229) | (0.325) | (0.073) | (0.102) | (0.094) | (0.134) |
| **Age (35-49)** |  |  |  |  |  |  |  |  |  |  |  |  |
| 16 to 19 | 2.157*** | -1.966** | 2.064*** | -2.598*** | 2.612*** | -2.880*** | 1.768** | -2.245* | 1.235*** | -1.115*** | 0.574* | -0.919* |
|  | (0.457) | (0.649) | (0.473) | (0.676) | (0.513) | (0.731) | (0.647) | (0.925) | (0.193) | (0.271) | (0.256) | (0.367) |
| 20 to 24 | 1.238*** | -0.678 | 0.997** | -0.732 | 1.094*** | -0.706 | 0.612 | -0.736 | 0.585*** | -0.379* | 0.207 | -0.247 |
|  | (0.296) | (0.446) | (0.304) | (0.460) | (0.317) | (0.481) | (0.363) | (0.557) | (0.101) | (0.152) | (0.145) | (0.223) |
| 25 to 34 | 0.298 | 0.257 | 0.216 | 0.151 | 0.376 | 0.048 | 0.279 | -0.156 | 0.301*** | -0.170 | 0.037 | 0.015 |
|  | (0.192) | (0.265) | (0.195) | (0.272) | (0.211) | (0.294) | (0.246) | (0.350) | (0.075) | (0.105) | (0.098) | (0.139) |
| 50 to 64 | 0.514** | -0.542* | 0.448** | -0.401 | 0.423* | -0.400 | 0.564** | -0.344 | -0.215** | 0.119 | 0.247** | -0.202 |
|  | (0.168) | (0.225) | (0.167) | (0.225) | (0.186) | (0.250) | (0.218) | (0.299) | (0.069) | (0.093) | (0.089) | (0.121) |
| 65 to 69 | 1.522*** | -1.304*** | 1.372*** | -1.293*** | 1.269*** | -0.961* | 1.309*** | -0.759 | 0.087 | -0.405* | 0.606*** | -0.488* |
|  | (0.293) | (0.386) | (0.294) | (0.389) | (0.322) | (0.427) | (0.381) | (0.516) | (0.127) | (0.168) | (0.153) | (0.206) |
| 70 to 74 | 3.052*** | -4.386*** | 2.934*** | -3.791*** | 2.166** | -2.808* | 0.744 | -0.368 | 0.215 | -0.653 | 1.049** | -1.424** |
|  | (0.669) | (0.997) | (0.674) | (1.010) | (0.739) | (1.107) | (0.906) | (1.371) | (0.272) | (0.408) | (0.357) | (0.538) |
| 75 + | 2.559*** | -3.919*** | 2.944*** | -4.068*** | 2.171** | -3.043** | 1.710 | -1.768 | 0.146 | -0.799 | 1.254*** | -1.794*** |
|  | (0.692) | (1.026) | (0.676) | (1.013) | (0.767) | (1.139) | (0.897) | (1.358) | (0.279) | (0.416) | (0.352) | (0.530) |
| Gender (Female =1) | 0.283* | -0.315 | 0.323** | -0.295 | 0.085 | -0.099 | -0.344* | 0.149 | -0.021 | 0.043 | -0.101 | -0.131 |
|  | (0.124) | (0.167) | (0.124) | (0.167) | (0.135) | (0.184) | (0.160) | (0.222) | (0.050) | (0.068) | (0.063) | (0.087) |
| Non-White Ethnicity | 0.258 | -0.479 | 0.155 | -0.235 | 0.202 | -0.320 | 0.227 | -0.531 | 0.141 | -0.223* | 0.052 | -0.248 |
|  | (0.188) | (0.253) | (0.190) | (0.257) | (0.204) | (0.277) | (0.239) | (0.333) | (0.076) | (0.104) | (0.099) | (0.136) |
| **Marital Status (Married)** |  |  |  |  |  |  |  |  |  |  |  |  |
| Single | -0.636*** | 0.450 | -0.821*** | 0.629** | -0.645*** | 0.447 | -0.190 | 0.199 | 0.047 | -0.007 | -0.349*** | 0.090 |
|  | (0.174) | (0.240) | (0.173) | (0.241) | (0.186) | (0.258) | (0.223) | (0.315) | (0.069) | (0.096) | (0.089) | (0.126) |
| Divorced/Separated | -0.482* | 0.073 | -0.760*** | 0.534* | -0.570** | 0.242 | -0.225 | 0.145 | -0.150 | 0.166 | -0.502*** | 0.077 |
|  | (0.198) | (0.268) | (0.195) | (0.265) | (0.215) | (0.293) | (0.251) | (0.348) | (0.081) | (0.111) | (0.099) | (0.136) |
| Widowed | -0.108 | -0.520 | -1.044** | 0.576 | -0.601 | 0.026 | 0.031 | -0.154 | -0.124 | 0.032 | -0.818*** | -0.032 |
|  | (0.367) | (0.503) | (0.389) | (0.532) | (0.418) | (0.570) | (0.451) | (0.623) | (0.154) | (0.210) | (0.181) | (0.249) |
| Missing | 0.040 | -0.571 | -0.082 | -0.137 | -0.006 | -0.314 | -0.102 | 0.300 | 0.169 | -0.341* | 0.043 | -0.209 |
|  | (0.288) | (0.438) | (0.282) | (0.429) | (0.302) | (0.463) | (0.373) | (0.582) | (0.110) | (0.167) | (0.147) | (0.232) |
| **Highest Qual (No educ)** |  |  |  |  |  |  |  |  |  |  |  |  |
| GCSEs | 0.454 | -1.670** | 0.533 | -1.229* | 0.209 | -1.014 | -0.200 | 0.147 | 0.235 | -0.318 | 0.010 | -0.381 |
|  | (0.358) | (0.598) | (0.356) | (0.602) | (0.396) | (0.663) | (0.460) | (0.791) | (0.140) | (0.241) | (0.182) | (0.313) |
| A Levels | 0.882 | -2.632*** | 0.764 | -1.856* | 0.258 | -1.429 | -0.446 | 0.239 | 0.345 | -0.551 | 0.095 | -0.702 |
|  | (0.512) | (0.798) | (0.526) | (0.822) | (0.576) | (0.899) | (0.673) | (1.072) | (0.203) | (0.321) | (0.260) | (0.416) |
| Degree | 0.859 | -2.906** | 1.031 | -2.391* | 0.447 | -1.947 | -0.847 | 0.405 | 0.609* | -0.803* | -0.024 | -0.725 |
|  | (0.658) | (0.988) | (0.665) | (1.002) | (0.731) | (1.101) | (0.851) | (1.307) | (0.257) | (0.392) | (0.331) | (0.507) |
| Log of Household Income | 0.905*** | -0.739*** | 0.822*** | -0.786*** | 0.882*** | -0.779*** | 0.575*** | -0.426* | 0.303*** | -0.226*** | 0.282*** | -0.219** |
|  | (0.108) | (0.149) | (0.108) | (0.150) | (0.118) | (0.163) | (0.139) | (0.196) | (0.043) | (0.060) | (0.056) | (0.079) |
| Number of Adults in Household | 0.470*** | -0.487*** | 0.396*** | -0.439*** | 0.440*** | -0.470*** | 0.299*** | -0.314** | 0.179*** | -0.179*** | 0.199*** | -0.138** |
|  | (0.065) | (0.092) | (0.067) | (0.094) | (0.072) | (0.102) | (0.083) | (0.121) | (0.027) | (0.039) | (0.034) | (0.049) |
| Number of Children in Household | 0.545*** | -0.777*** | 0.611*** | -0.747*** | 0.563*** | -0.798*** | 0.420** | -0.611** | 0.237*** | -0.268*** | 0.166** | -0.218** |
|  | (0.112) | (0.152) | (0.114) | (0.154) | (0.124) | (0.169) | (0.149) | (0.205) | (0.045) | (0.062) | (0.060) | (0.082) |
| Homeowner | 0.441** | -0.479* | 0.557*** | -0.696** | 0.428* | -0.502 | 0.482* | -0.596 | 0.363*** | -0.395*** | 0.163 | -0.100 |
|  | (0.168) | (0.242) | (0.168) | (0.244) | (0.186) | (0.269) | (0.219) | (0.320) | (0.068) | (0.097) | (0.089) | (0.129) |
| **IMD (IMD=3)** |  |  |  |  |  |  |  |  |  |  |  |  |
| 1 - Most | -0.216 | 0.321 | -0.147 | 0.278 | -0.175 | 0.284 | 0.273 | -0.356 | -0.067 | 0.011 | 0.114 | -0.162 |
|  | (0.184) | (0.265) | (0.188) | (0.272) | (0.204) | (0.296) | (0.240) | (0.356) | (0.077) | (0.113) | (0.097) | (0.143) |
| 2 | 0.109 | -0.260 | -0.045 | 0.003 | 0.169 | -0.302 | 0.264 | -0.406 | 0.077 | -0.143 | 0.134 | -0.200 |
|  | (0.175) | (0.244) | (0.176) | (0.246) | (0.191) | (0.266) | (0.221) | (0.315) | (0.069) | (0.098) | (0.090) | (0.127) |
| 4 | 0.037 | -0.102 | -0.063 | 0.056 | 0.194 | -0.297 | 0.057 | -0.177 | 0.043 | -0.034 | -0.013 | -0.026 |
|  | (0.186) | (0.247) | (0.188) | (0.249) | (0.204) | (0.273) | (0.250) | (0.338) | (0.077) | (0.103) | (0.099) | (0.133) |
| 5 - Least | 0.264 | -0.428 | 0.333 | -0.514* | 0.144 | -0.256 | -0.062 | -0.029 | 0.296*** | -0.337** | 0.118 | -0.237 |
|  | (0.198) | (0.259) | (0.200) | (0.261) | (0.226) | (0.295) | (0.273) | (0.362) | (0.080) | (0.106) | (0.108) | (0.141) |
| **Region (London Base)** |  |  |  |  |  |  |  |  |  |  |  |  |
| North East | 0.012 | 0.529 | -0.218 | 0.654 | -0.350 | 0.799 | 0.139 | 0.334 | -0.132 | 0.132 | 0.081 | 0.088 |
|  | (0.289) | (0.409) | (0.290) | (0.412) | (0.320) | (0.454) | (0.380) | (0.554) | (0.117) | (0.167) | (0.150) | (0.216) |
| North West | 0.187 | 0.089 | -0.146 | 0.475 | 0.121 | 0.145 | -0.092 | 0.631 | -0.172* | 0.215* | -0.120 | 0.301* |
|  | (0.195) | (0.264) | (0.202) | (0.275) | (0.214) | (0.293) | (0.257) | (0.358) | (0.078) | (0.109) | (0.103) | (0.142) |
| Yorkshire and Humberside | 0.205 | 0.031 | -0.123 | 0.373 | -0.208 | 0.513 | 0.003 | 0.515 | -0.167 | 0.211 | -0.084 | 0.253 |
|  | (0.217) | (0.299) | (0.213) | (0.294) | (0.231) | (0.318) | (0.278) | (0.392) | (0.087) | (0.122) | (0.115) | (0.160) |
| East Midlands | 0.469* | -0.304 | 0.003 | 0.196 | 0.400 | -0.365 | 0.610* | -0.478 | -0.207* | 0.223 | -0.006 | 0.090 |
|  | (0.225) | (0.310) | (0.233) | (0.324) | (0.251) | (0.349) | (0.300) | (0.425) | (0.093) | (0.129) | (0.122) | (0.171) |
| West Midlands | 0.222 | -0.046 | -0.420* | 0.797** | -0.025 | 0.193 | 0.066 | 0.293 | -0.040 | 0.007 | -0.004 | 0.085 |
|  | (0.203) | (0.276) | (0.205) | (0.278) | (0.219) | (0.300) | (0.262) | (0.364) | (0.077) | (0.107) | (0.105) | (0.146) |
| East of England | -0.083 | 0.354 | -0.413 | 0.756* | -0.029 | 0.197 | -0.138 | 0.499 | -0.279*** | 0.343** | -0.260* | 0.478** |
|  | (0.218) | (0.296) | (0.220) | (0.298) | (0.239) | (0.325) | (0.273) | (0.379) | (0.084) | (0.115) | (0.109) | (0.150) |
| South East | -0.265 | 0.466 | -0.347 | 0.570* | -0.523* | 0.719** | -0.285 | 0.721* | -0.229** | 0.247* | -0.236* | 0.345** |
|  | (0.190) | (0.252) | (0.190) | (0.253) | (0.208) | (0.277) | (0.244) | (0.332) | (0.072) | (0.097) | (0.098) | (0.133) |
| South West | 0.347 | -0.147 | 0.104 | 0.108 | 0.052 | 0.160 | -0.262 | 0.814* | -0.208* | 0.274* | -0.096 | 0.208 |
|  | (0.230) | (0.302) | (0.233) | (0.309) | (0.246) | (0.326) | (0.292) | (0.394) | (0.096) | (0.128) | (0.123) | (0.164) |
| Rural LSOA | -0.148 | 0.178 | -0.071 | 0.131 | -0.277 | 0.346 | 0.226 | -0.327 | 0.085 | -0.083 | -0.063 | 0.029 |
|  | (0.220) | (0.285) | (0.221) | (0.286) | (0.234) | (0.304) | (0.272) | (0.358) | (0.083) | (0.108) | (0.110) | (0.144) |
| **Survey Year (2013)** |  |  |  |  |  |  |  |  |  |  |  |  |
| 2014 | 0.067 | 0.117 | -0.399 | 0.535 | -0.072 | 0.224 | 0.659 | -0.811 | 0.124 | -0.113 | 0.222 | -0.240 |
|  | (0.359) | (0.453) | (0.367) | (0.463) | (0.384) | (0.486) | (0.448) | (0.575) | (0.149) | (0.190) | (0.191) | (0.245) |
| 2015 | -0.237 | 0.696 | -0.650 | 1.041* | -0.411 | 0.894 | -0.070 | 0.355 | 0.034 | -0.053 | -0.409* | 0.573* |
|  | (0.361) | (0.461) | (0.368) | (0.467) | (0.381) | (0.490) | (0.473) | (0.616) | (0.137) | (0.177) | (0.196) | (0.254) |
| 2016 | 0.160 | 0.153 | -0.489 | 0.680 | 0.147 | 0.107 | -0.165 | 0.283 | -0.162 | 0.229 | -0.132 | 0.200 |
|  | (0.277) | (0.357) | (0.289) | (0.370) | (0.306) | (0.396) | (0.369) | (0.479) | (0.108) | (0.141) | (0.143) | (0.185) |
| 2017 | 0.392 | -0.057 | -0.194 | 0.521 | 0.410 | -0.136 | 0.539 | -0.553 | 0.033 | 0.056 | 0.242 | -0.242 |
|  | (0.277) | (0.359) | (0.283) | (0.366) | (0.307) | (0.399) | (0.364) | (0.478) | (0.107) | (0.141) | (0.144) | (0.189) |
| 2018 | 0.489 | -0.231 | -0.069 | 0.287 | 0.391 | -0.122 | 0.363 | -0.434 | 0.045 | 0.001 | 0.101 | -0.090 |
|  | (0.282) | (0.369) | (0.286) | (0.372) | (0.310) | (0.407) | (0.373) | (0.494) | (0.107) | (0.142) | (0.144) | (0.190) |
| 2019 | 0.385 | -0.103 | -0.101 | 0.331 | 0.681* | -0.574 | 0.070 | -0.294 | -0.070 | 0.117 | 0.217 | -0.289 |
|  | (0.268) | (0.347) | (0.271) | (0.350) | (0.293) | (0.382) | (0.353) | (0.464) | (0.104) | (0.137) | (0.138) | (0.181) |
| 2020 | 0.351 | 0.060 | -0.242 | 0.632 | 0.400 | -0.174 | 0.289 | -0.606 | -0.012 | 0.174 | 0.136 | -0.080 |
|  | (0.402) | (0.553) | (0.398) | (0.549) | (0.443) | (0.613) | (0.529) | (0.744) | (0.157) | (0.219) | (0.205) | (0.287) |
| Constant | -3.436*** | 11.953*** | -1.018 | 10.427*** | -2.757** | 11.151*** | -0.942 | 6.828** | 0.563 | 3.197*** | 0.467 | 3.591*** |
|  | (0.983) | (1.599) | (0.973) | (1.614) | (1.053) | (1.754) | (1.213) | (2.115) | (0.382) | (0.639) | (0.494) | (0.859) |
| Observations | 50,006 | | 50,006 | | 50,006 | | 50,006 | | 50,006 | | 50,006 | |

**Note:** Robust standard errors in parentheses, * p<0.05, ** p<0.01, *** p<0.001, models adjusted using population weights.

Table A 4: Parameters from the Marginal Treatment Effects model using unweighted sample with Bootstrapped and Robust Standard errors

|  | (1) | | (2) | | (3) | | (4) | | (5) | | (6) | |
| --- | --- | --- | --- | --- | --- | --- | --- | --- | --- | --- | --- | --- |
|  | **Life Satisfaction** | | **Worthwhile** | | **Happiness** | | **Anxiety** | | **Health** | | **Lonely** | |
|  | Robust | Bootstrap | Robust | Bootstrap | Robust | Bootstrap | Robust | Bootstrap | Robust | Bootstrap | Robust | Bootstrap |
| ATE | 1.733*** | 1.733*** | 1.561*** | 1.561*** | 1.526*** | 1.526*** | 1.821*** | 1.821*** | 0.300* | 0.300* | 0.683*** | 0.683*** |
|  | (0.331) | (0.343) | (0.339) | (0.355) | (0.368) | (0.384) | (0.447) | (0.451) | (0.134) | (0.138) | (0.181) | (0.185) |
| ATT | 0.794 | 0.794 | 0.647 | 0.647 | 0.776 | 0.776 | 1.596* | 1.596** | -0.082 | -0.082 | 0.367 | 0.367 |
|  | (0.484) | (0.510) | (0.487) | (0.505) | (0.531) | (0.570) | (0.625) | (0.613) | (0.191) | (0.196) | (0.254) | (0.248) |
| ATU | 3.482*** | 3.482*** | 3.267*** | 3.267*** | 2.923*** | 2.923*** | 2.210** | 2.210** | 1.019*** | 1.019*** | 1.265*** | 1.265*** |
|  | (0.464) | (0.497) | (0.482) | (0.497) | (0.529) | (0.537) | (0.686) | (0.692) | (0.196) | (0.205) | (0.270) | (0.278) |
| LATE | 2.360*** | 2.360*** | 2.250*** | 2.250*** | 1.991*** | 1.991*** | 1.863*** | 1.863*** | 0.591*** | 0.591*** | 0.818*** | 0.818*** |
|  | (0.307) | (0.347) | (0.321) | (0.355) | (0.350) | (0.372) | (0.454) | (0.470) | (0.130) | (0.144) | (0.180) | (0.192) |
| 1st MPRTE | 0.247*** | 0.247*** | 0.232*** | 0.232*** | 0.208*** | 0.208*** | 0.163** | 0.163** | 0.071*** | 0.071*** | 0.091*** | 0.091*** |
|  | (0.034) | (0.036) | (0.035) | (0.037) | (0.038) | (0.039) | (0.050) | (0.051) | (0.014) | (0.015) | (0.020) | (0.021) |
| 2nd MPRTE | 0.265*** | 0.265*** | 0.247*** | 0.247*** | 0.223*** | 0.223*** | 0.178*** | 0.178** | 0.076*** | 0.076*** | 0.098*** | 0.098*** |
|  | (0.036) | (0.039) | (0.038) | (0.040) | (0.042) | (0.042) | (0.054) | (0.055) | (0.015) | (0.016) | (0.021) | (0.022) |
| 3rd MPRTE | 0.303*** | 0.303*** | 0.283*** | 0.283*** | 0.254*** | 0.254*** | 0.199** | 0.199** | 0.088*** | 0.088*** | 0.112*** | 0.112*** |
|  | (0.042) | (0.046) | (0.044) | (0.046) | (0.048) | (0.049) | (0.063) | (0.064) | (0.018) | (0.019) | (0.025) | (0.026) |
| Observations | 50,028 | 50,028 | 50,028 | 50,028 | 50,028 | 50,028 | 50,028 | 50,028 | 50,028 | 50,028 | 50,028 | 50,028 |

**Note:** Standard errors in parentheses, * p<0.05, ** p<0.01, *** p<0.001. Models include individual, household, area and asset controls, see table A 1. ATE – average treatment effect, ATT- average treatment effect on treated, ATU – average treatment effect on untreated, LATE – local average treatment effect, MPRTE – marginal policy relevant treatment effect. 1^st^ indicates an incremental shift in the instrument, 2^nd^ indicates an incremental shift in the absolute value of the propensity scores, and 3^rd^ indicates an incremental shift in the relative value of the propensity scores (see table 4)

Table A 5 Average Marginal Effects for First Stage Results with test for relevance and monotonicity for sensitivity analysis

|  | (1) | (2) | (3) | (4) |
| --- | --- | --- | --- | --- |
|  | **Participate**  No Sport and Exercise groups | | **Participate**  No Trade Unions or Children's  Education and Activities | |
| **Binary Assets** |  |  |  |  |
| Church/place of worship | 0.067*** |  | 0.067*** |  |
|  | (0.052 to 0.082) |  | (0.052 to 0.082) |  |
| Community centre/hall | 0.035*** |  | 0.032*** |  |
|  | (0.024 to 0.047) |  | (0.021 to 0.043) |  |
| **Count of Assets (base=0)** |  |  |  |  |
| 1 |  | 0.072*** |  | 0.069*** |
|  |  | (0.053 to 0.091) |  | (0.051 to 0.088) |
| 2 |  | 0.108*** |  | 0.104*** |
|  |  | (0.088 to 0.127) |  | (0.085 to 0.123) |
| Wald’s chi-squared score (Relevance) | 122.348 | 121.682 | 124.382 | 119.424 |
| p-value | <0.001 | <0.001 | <0.001 | <0.001 |
| Observations | 50,041 | 50,041 | 49,975 | 49,975 |

**Note:** Robust standard errors in parentheses, * p<0.05, ** p<0.01, *** p<0.001, models adjusted using population weights. Models include individual, household, area and asset controls, see table A 1. Wald’s Chi-squared test has chi-squared distribution and degrees of freedom equal to number of instruments (Z_= 2) (Gregory and Veall 1985)

Table A 6: Endogeneity and Overidentifying Restrictions for the outcomes for sensitivity analysis

|  | (1) | | (2) | (3) | (4) | (5) | (6) |
| --- | --- | --- | --- | --- | --- | --- | --- |
|  | Life Satisfaction | | Worthwhile | Happiness | Anxiety | Health | Lonely |
| **No Sport and Exercise groups** | | | | | | | |
| **Robust Test Score**  (Endogeneity) | 53.497 | 31.415 | | 35.795 | 23.244 | 8.729 | 23.307 |
| p-value | <0.001 | <0.001 | | <0.001 | <0.001 | 0.003 | <0.001 |
| **Sargan’s chi-squared score**  (Overidentifying Restrictions) | 0.531 | 0.308 | | 2.135 | 0.027 | 1.537 | 2.838 |
| p-value | 0.466 | 0.579 | | 0.144 | 0.870 | 0.215 | 0.092 |
| **No Trade Unions or Children's Education and Activities** | | | | | | | |
| **Robust test score**  (Endogeneity) | 51.004 | 30.754 | | 33.003 | 22.592 | 5.933 | 20.988 |
| p-value | <0.001 | <0.001 | | <0.001 | <0.001 | 0.015 | <0.001 |
| **Sargan’s chi-squared sore**  (Overidentifying Restrictions) | 1.285 | 0.866 | | 3.288 | 0.013 | 2.453 | 3.531 |
| p-value | 0.257 | 0.352 | | 0.070 | 0.908 | 0.117 | 0.060 |
|  |  |  | |  |  |  |  |

**Note:** Robust standard errors in parentheses, * p<0.05, ** p<0.01, *** p<0.001, models adjusted using population weights. Models include individual, household, area and asset controls, see table A 1. Robust test score has a chi-squared distribution and degrees of freedom equal to number of endogenous regressors (k=1) (Wooldridge 1995). Sargan’s chi-square test has a chi-squared distribution and degrees of freedom equal to number of instruments minus number of endogenous regressors (Z_- k =1) (Sargan 1958). Number of observations equal to 50,041 for “No Sport and Exercise groups”, and equal to 49,975 for “No Trade Unions or Children's Education and Activities”

Table A 7: Marginal Treatment Effects heterogeneity tests and treatment effect parameters for social participation without sports and exercise groups attendance

|  | (1) | (2) | (3) | (4) | (5) | (6) |
| --- | --- | --- | --- | --- | --- | --- |
|  | Life Satisfaction | Worthwhile | Happiness | Anxiety | Health | Lonely |
| ATE | 2.107*** | 1.733*** | 2.023*** | 1.464** | 0.414** | 0.914*** |
|  | (0.374) | (0.390) | (0.421) | (0.513) | (0.151) | (0.207) |
| ATT | 1.225* | 0.685 | 1.501* | 0.805 | 0.106 | 0.727* |
|  | (0.572) | (0.590) | (0.642) | (0.757) | (0.228) | (0.304) |
| ATU | 3.344*** | 3.203*** | 2.755*** | 2.383** | 0.844*** | 1.175*** |
|  | (0.540) | (0.566) | (0.612) | (0.774) | (0.222) | (0.303) |
| LATE | 2.469*** | 2.114*** | 2.189*** | 1.620** | 0.494** | 0.844*** |
|  | (0.370) | (0.388) | (0.418) | (0.520) | (0.153) | (0.208) |
| 1st MPRTE | 0.796*** | 0.764*** | 0.657*** | 0.576** | 0.203*** | 0.281*** |
|  | (0.132) | (0.138) | (0.149) | (0.189) | (0.054) | (0.074) |
| 2nd MPRTE | 0.816*** | 0.781*** | 0.670*** | 0.592** | 0.207*** | 0.288*** |
|  | (0.136) | (0.143) | (0.154) | (0.195) | (0.056) | (0.076) |
| 3rd MPRTE | 0.969*** | 0.937*** | 0.788*** | 0.705** | 0.250*** | 0.337*** |
|  | (0.168) | (0.175) | (0.190) | (0.239) | (0.069) | (0.094) |
| Inverse Mills  ratio | 3.808* | 4.535** | 2.229 | 2.965 | 1.345* | 0.819 |
|  | (1.572) | (1.621) | (1.770) | (2.149) | (0.632) | (0.840) |
| **P-Value** |  |  |  |  |  |  |
| Observed Heterogeneity | <0.001 | <0.001 | <0.001 | <0.001 | 0.015 | <0.001 |
| Essential Heterogeneity | 0.015 | 0.005 | 0.208 | 0.168 | 0.033 | 0.329 |
| Observations | 50,041 | 50,041 | 50,041 | 50,041 | 50,041 | 50,041 |

**Note:** Robust standard errors in parentheses, * p<0.05, ** p<0.01, *** p<0.001, models adjusted using population weights. Models include individual, household, area and asset controls, see table A 1. ATE – average treatment effect, ATT- average treatment effect on treated, ATU – average treatment effect on untreated, LATE – local average treatment effect, MPRTE – marginal policy relevant treatment effect. 1^st^ indicates an incremental shift in the instrument, 2^nd^ indicates an incremental shift in the absolute value of the propensity scores, and 3^rd^ indicates an incremental shift in the relative value of the propensity scores (see table 4)

Table A 8: Marginal Treatment Effects heterogeneity tests and treatment effect parameters for social participation without trade union, children’s groups and youth activities attendance

|  | (1) | (2) | (3) | (4) | (5) | (6) |
| --- | --- | --- | --- | --- | --- | --- |
|  | Life Satisfaction | Worthwhile | Happiness | Anxiety | Health | Lonely |
| ATE | 2.277*** | 1.903*** | 2.126*** | 1.539** | 0.456** | 0.928*** |
|  | (0.401) | (0.416) | (0.450) | (0.544) | (0.165) | (0.219) |
| ATT | 1.000 | 0.703 | 0.996 | 1.089 | 0.071 | 0.569 |
|  | (0.610) | (0.615) | (0.678) | (0.787) | (0.242) | (0.313) |
| ATU | 4.426*** | 3.924*** | 4.028*** | 2.284** | 1.106*** | 1.530*** |
|  | (0.581) | (0.608) | (0.661) | (0.837) | (0.241) | (0.329) |
| LATE | 3.352*** | 2.936*** | 3.078*** | 1.913** | 0.782*** | 1.079*** |
|  | (0.405) | (0.429) | (0.460) | (0.586) | (0.171) | (0.234) |
| 1st MPRTE | 0.486*** | 0.431*** | 0.443*** | 0.259** | 0.122*** | 0.170*** |
|  | (0.064) | (0.067) | (0.073) | (0.093) | (0.027) | (0.036) |
| 2nd MPRTE | 0.512*** | 0.452*** | 0.466*** | 0.274** | 0.128*** | 0.180*** |
|  | (0.069) | (0.072) | (0.078) | (0.099) | (0.028) | (0.039) |
| 3rd MPRTE | 0.592*** | 0.524*** | 0.539*** | 0.313** | 0.149*** | 0.206*** |
|  | (0.081) | (0.085) | (0.092) | (0.117) | (0.034) | (0.046) |
| Inverse Mills  ratio | 6.454*** | 6.025*** | 5.720** | 2.475 | 1.951** | 1.878* |
|  | (1.758) | (1.774) | (1.971) | (2.357) | (0.701) | (0.916) |
| **P-Value** |  |  |  |  |  |  |
| Observed Heterogeneity | <0.001 | <0.001 | <0.001 | <0.001 | <0.001 | <0.001 |
| Essential Heterogeneity | <0.001 | 0.001 | 0.004 | 0.294 | 0.005 | 0.040 |
| Observations | 49,975 | 49,975 | 49,975 | 49,975 | 49,975 | 49,975 |

**Note:** Robust standard errors in parentheses, * p<0.05, ** p<0.01, *** p<0.001, models adjusted using population weights. Models include individual, household, area and asset controls, see table A 1. ATE – average treatment effect, ATT- average treatment effect on treated, ATU – average treatment effect on untreated, LATE – local average treatment effect, MPRTE – marginal policy relevant treatment effect. 1^st^ indicates an incremental shift in the instrument, 2^nd^ indicates an incremental shift in the absolute value of the propensity scores, and 3^rd^ indicates an incremental shift in the relative value of the propensity scores (see table 4)

Table A 9: Estimated average marginal effects and tests of instrument relevance for each potential instrument included individually

|  | Church/place of worship | Community centre/hall | Sports centre/facility/club | Youth club/centre/facility | Park | Public transport links |
| --- | --- | --- | --- | --- | --- | --- |
| AME on social participation | 0.063*** | 0.031*** | 0.041*** | 0.021*** | 0.016* | 0.019* |
| 95% confidence interval | (0.048 to 0.077) | (0.020 to 0.041) | (0.031 to 0.052) | (0.009 to 0.033) | (0.001 to 0.031) | (0.000 to 0.038) |
|  |  |  |  |  |  |  |
| Wald’s chi-squared score | 74.145 | 30.506 | 57.646 | 11.764 | 3.874 | 4.102 |
| p-value | <0.001 | <0.001 | <0.001 | 0.001 | 0.049 | 0.043 |
| Observations | 50,006 | 50,006 | 50,006 | 50,006 | 50,006 | 50,006 |

**Note:** 95% Confidence Intervals based on robust standard errors in parentheses, * p<0.05, ** p<0.01, *** p<0.001, models adjusted using population weights. Models include individual, household, area and asset controls, see table A 1.

Table A 10: Overidentifying Restrictions test via Sargan's chi-squared score and p-value for outcomes with different instrument choice

|  |  |  |  |  | (1) | (2) | (3) | (4) | (5) | (6) |
| --- | --- | --- | --- | --- | --- | --- | --- | --- | --- | --- |
|  | **Assets as Instruments** | | | | Life Satisfaction | Worthwhile | Happiness | Anxiety | Health | Lonely |
| **Main Model** | Place of worship | Community centre |  |  | 1.221 | 0.832 | 3.104 | 0.021 | 2.510 | 3.580 |
|  |  |  |  |  | 0.269 | 0.362 | 0.078 | 0.885 | 0.113 | 0.058 |
| **Group:** |  |  |  |  |  |  |  |  |  |  |
| **1** | Place of worship | Community centre | Sports centre | Youth club | 16.891 | 15.193 | 20.548 | 3.173 | 25.428 | 13.101 |
|  |  |  |  |  | 0.001 | 0.002 | <0.001 | 0.366 | <0.001 | 0.004 |
| **2** | Place of worship | Community centre | Sports centre |  | 2.951 | 3.020 | 6.123 | 0.134 | 25.550 | 3.728 |
|  |  |  |  |  | 0.229 | 0.221 | 0.047 | 0.935 | <0.001 | 0.155 |
| **3** | Place of worship | Community centre | Youth club |  | 16.887 | 14.628 | 19.779 | 2.955 | 2.995 | 13.181 |
|  |  |  |  |  | <0.001 | 0.001 | <0.001 | 0.228 | 0.224 | 0.001 |
| **4** | Place of worship | Sports centre | Youth club |  | 17.113 | 15.299 | 20.740 | 3.151 | 25.019 | 11.600 |
|  |  |  |  |  | <0.001 | <0.001 | <0.001 | 0.207 | <0.001 | 0.003 |
| **5** | Community centre | Sports centre | Youth club |  | 7.233 | 6.387 | 6.048 | 2.836 | 6.265 | 5.382 |
|  |  |  |  |  | 0.027 | 0.041 | 0.049 | 0.242 | 0.044 | 0.068 |
| **6** | Place of worship | Sports centre |  |  | 2.801 | 2.994 | 5.544 | 0.068 | 25.157 | 1.316 |
|  |  |  |  |  | 0.094 | 0.084 | 0.019 | 0.794 | <0.001 | 0.251 |
| **7** | Place of worship | Youth club |  |  | 17.219 | 14.733 | 19.274 | 2.961 | 0.691 | 11.277 |
|  |  |  |  |  | <0.001 | <0.001 | 0.000 | 0.085 | 0.406 | 0.001 |
| **8** | Community centre | Sports centre |  |  | 0.040 | 0.194 | 0.010 | 0.118 | 4.615 | 0.879 |
|  |  |  |  |  | 0.842 | 0.660 | 0.919 | 0.732 | 0.032 | 0.348 |
| **9** | Community centre | Youth club |  |  | 5.637 | 5.550 | 4.661 | 1.779 | 0.038 | 2.165 |
|  |  |  |  |  | 0.018 | 0.018 | 0.031 | 0.182 | 0.845 | 0.141 |
| **10** | Sports centre | Youth club |  |  | 6.417 | 5.301 | 5.490 | 2.837 | 2.195 | 5.318 |
|  |  |  |  |  | 0.011 | 0.021 | 0.019 | 0.092 | 0.138 | 0.021 |

**Note:** Models adjusted using population weights. Models include individual, household, area and asset controls. Green shading indicators failure to reject the null hypothesis – instrument choice is valid.

Table A 11: Average Treatment Effects from Marginal Treatment Effects Model with different instrument choice

|  |  |  |  | |  | (1) | (2) | (3) | (4) | (5) | (6) |
| --- | --- | --- | --- | --- | --- | --- | --- | --- | --- | --- | --- |
|  | **Assets as Instruments** | | | | | Life Satisfaction | Worthwhile | Happiness | Anxiety | Health | Lonely |
| **Main Model** | Place of worship | Community centre |  | |  | 2.173*** | 1.704*** | 2.032*** | 1.500** | 0.403* | 0.966*** |
|  |  |  |  | |  | (0.429) | (0.442) | (0.479) | (0.576) | (0.175) | (0.231) |
| **Group:** |  |  |  | |  |  |  |  |  |  |  |
| **1** | Place of worship | Community centre | Sports centre | Youth club | | 2.530*** | 2.226*** | 2.592*** | 1.565*** | 0.660*** | 0.960*** |
|  |  |  |  | |  | (0.264) | (0.274) | (0.298) | (0.364) | (0.109) | (0.146) |
| **2** | Place of worship | Community centre | Sports centre | |  | 2.074*** | 1.759*** | 2.076*** | 1.335*** | 0.687*** | 0.834*** |
|  |  |  |  | |  | (0.297) | (0.306) | (0.335) | (0.404) | (0.122) | (0.161) |
| **3** | Place of worship | Community centre | Youth club | |  | 2.296*** | 1.950*** | 2.225*** | 1.302** | 0.337* | 0.945*** |
|  |  |  |  | |  | (0.325) | (0.339) | (0.364) | (0.446) | (0.135) | (0.181) |
| **4** | Place of worship | Sports centre | Youth club | |  | 2.412*** | 2.163*** | 2.338*** | 1.453*** | 0.682*** | 0.880*** |
|  |  |  |  | |  | (0.313) | (0.321) | (0.353) | (0.424) | (0.127) | (0.173) |
| **5** | Community centre | Sports centre | Youth club | |  | 2.758*** | 2.517*** | 3.073*** | 1.433** | 0.806*** | 1.092*** |
|  |  |  |  | |  | (0.345) | (0.357) | (0.380) | (0.461) | (0.140) | (0.187) |
| **6** | Place of worship | Sports centre |  | |  | 1.976*** | 1.717*** | 1.847*** | 1.224** | 0.704*** | 0.758*** |
|  |  |  |  | |  | (0.338) | (0.345) | (0.382) | (0.456) | (0.138) | (0.185) |
| **7** | Place of worship | Youth club |  | |  | 2.048*** | 1.767*** | 1.764*** | 1.047* | 0.269 | 0.821*** |
|  |  |  |  | |  | (0.392) | (0.404) | (0.438) | (0.528) | (0.161) | (0.218) |
| **8** | Community centre | Sports centre |  | |  | 2.091*** | 1.836*** | 2.383*** | 1.040 | 0.899*** | 0.937*** |
|  |  |  |  | |  | (0.400) | (0.414) | (0.444) | (0.534) | (0.163) | (0.217) |
| **9** | Community centre | Youth club |  | |  | 2.475*** | 2.189*** | 2.743*** | 0.757 | 0.282 | 1.163*** |
|  |  |  |  | |  | (0.492) | (0.502) | (0.538) | (0.648) | (0.202) | (0.266) |
| **10** | Sports centre | Youth club |  | |  | 2.704*** | 2.569*** | 2.897*** | 1.289* | 0.906*** | 1.033*** |
|  |  |  |  | |  | (0.431) | (0.439) | (0.473) | (0.563) | (0.172) | (0.230) |

**Note:** Number of observation is 50,006. Robust standard errors in parentheses, * p<0.05, ** p<0.01, *** p<0.001, models adjusted using population weights. Models include individual, household, area and asset controls, see table A 1. Green shading indicators failure to reject the null hypothesis – instrument choice is valid.

Table A 12: Marginal Treatment Effects heterogeneity tests and treatment effect parameters excluding individuals who reside in rural area residents

|  | (1) | (2) | (3) | (4) | (5) | (6) |
| --- | --- | --- | --- | --- | --- | --- |
|  | Life Satisfaction | Worthwhile | Happiness | Anxiety | Health | Lonely |
| ATE | 1.179*** | 1.132*** | 1.087** | 1.154** | 0.139 | 0.405* |
|  | (0.307) | (0.316) | (0.340) | (0.415) | (0.124) | (0.168) |
| ATT | 0.578 | 0.591 | 0.763 | 1.289* | -0.120 | 0.233 |
|  | (0.441) | (0.445) | (0.480) | (0.560) | (0.172) | (0.230) |
| ATU | 2.297*** | 2.142*** | 1.698** | 0.891 | 0.627*** | 0.722** |
|  | (0.458) | (0.476) | (0.522) | (0.671) | (0.191) | (0.266) |
| LATE | 1.628*** | 1.587*** | 1.294*** | 0.936* | 0.345** | 0.472** |
|  | (0.303) | (0.317) | (0.345) | (0.444) | (0.127) | (0.177) |
| 1st MPRTE | 0.287*** | 0.270*** | 0.221*** | 0.143 | 0.070** | 0.093** |
|  | (0.055) | (0.058) | (0.063) | (0.082) | (0.023) | (0.032) |
| 2nd MPRTE | 0.296*** | 0.276*** | 0.223*** | 0.146 | 0.072** | 0.096** |
|  | (0.059) | (0.062) | (0.067) | (0.087) | (0.025) | (0.034) |
| 3rd MPRTE | 0.341*** | 0.316*** | 0.252** | 0.153 | 0.086** | 0.110** |
|  | (0.070) | (0.073) | (0.080) | (0.103) | (0.029) | (0.041) |
| Inverse Mills | 2.633* | 2.338* | 1.359 | -0.498 | 1.091* | 0.791 |
| Ratio | (1.108) | (1.123) | (1.229) | (1.493) | (0.442) | (0.600) |
| **P-Value** |  |  |  |  |  |  |
| Observed Heterogeneity | <0.001 | <0.001 | <0.001 | <0.001 | <0.001 | <0.001 |
| Essential Heterogeneity | 0.017 | 0.037 | 0.269 | 0.739 | 0.013 | 0.188 |
| Observations | 41,171 | 41,171 | 41,171 | 41,171 | 41,171 | 41,171 |

**Note:** Robust standard errors in parentheses, * p<0.05, ** p<0.01, *** p<0.001, models adjusted using population weights. Models include individual, household, area and asset controls, see table A 1. ATE – average treatment effect, ATT- average treatment effect on treated, ATU – average treatment effect on untreated, LATE – local average treatment effect, MPRTE – marginal policy relevant treatment effect. 1^st^ indicates an incremental shift in the instrument, 2^nd^ indicates an incremental shift in the absolute value of the propensity scores, and 3^rd^ indicates an incremental shift in the relative value of the propensity scores (see table 4)

Table A 13: First stage regression results excluding individuals with long term conditions that limit day to day activities

|  | (1) | (2) |
| --- | --- | --- |
|  |  |  |
| **Binary Assets** |  |  |
| Church/place of worship | 0.079*** |  |
|  | (0.067 to 0.091) |  |
| Community centre/hall | 0.034*** |  |
|  | (0.025 to 0.043) |  |
| **Count of Assets (base=0)** |  |  |
| 1 |  | 0.082*** |
|  |  | (0.066 to 0.097) |
| 2 |  | 0.120*** |
|  |  | (0.104 to 0.136) |
| **Wald’s chi-squared score**  (Relevance) | 252.550 | 235.838 |
| p-value | <0.001 | <0.001 |
| Observations | 45751 | 45751 |

**Note:** 95% Confidence Intervals based on robust standard errors in parentheses, * p<0.05, ** p<0.01, *** p<0.001, models adjusted using population weights. Models include individual, household, area and asset controls, see table A 1. Wald’s Chi-squared test has chi-squared distribution and degrees of freedom equal to number of instruments (Z_= 2) (Gregory and Veall 1985)

Table A 14: Marginal Treatment Effects heterogeneity tests and treatment effect parameters excluding individuals with long term conditions that limit day to day activities

|  | (1) | (2) | (3) | (4) | (5) | (6) |
| --- | --- | --- | --- | --- | --- | --- |
|  | Life Satisfaction | Worthwhile | Happiness | Anxiety | Health | Lonely |
| ATE | 1.212*** | 1.168*** | 1.171*** | 1.416*** | 0.239* | 0.472** |
|  | (0.281) | (0.285) | (0.313) | (0.389) | (0.107) | (0.157) |
| ATT | 0.639 | 0.677 | 0.661 | 1.248* | 0.045 | 0.254 |
|  | (0.393) | (0.393) | (0.433) | (0.524) | (0.146) | (0.213) |
| ATU | 2.377*** | 2.173*** | 2.203*** | 1.726** | 0.638*** | 0.910*** |
|  | (0.364) | (0.379) | (0.417) | (0.553) | (0.146) | (0.217) |
| LATE | 1.685*** | 1.640*** | 1.565*** | 1.475*** | 0.395*** | 0.584*** |
|  | (0.256) | (0.267) | (0.294) | (0.387) | (0.102) | (0.153) |
| 1st MPRTE | 0.235*** | 0.215*** | 0.221*** | 0.188*** | 0.061*** | 0.091*** |
|  | (0.035) | (0.037) | (0.040) | (0.053) | (0.014) | (0.021) |
| 2nd MPRTE | 0.240*** | 0.218*** | 0.226*** | 0.198*** | 0.061*** | 0.095*** |
|  | (0.036) | (0.038) | (0.042) | (0.055) | (0.015) | (0.022) |
| 3rd MPRTE | 0.271*** | 0.246*** | 0.255*** | 0.217*** | 0.070*** | 0.107*** |
|  | (0.042) | (0.044) | (0.048) | (0.064) | (0.017) | (0.025) |
| Inverse Mills | 3.000** | 2.527** | 2.729** | 1.217 | 0.978** | 1.203* |
| Ratio | (0.946) | (0.953) | (1.059) | (1.326) | (0.362) | (0.529) |
| **P-Value** |  |  |  |  |  |  |
| Observed Heterogeneity | <0.001 | <0.001 | <0.001 | 0.010 | <0.001 | <0.001 |
| Essential Heterogeneity | 0.002 | 0.008 | 0.010 | 0.359 | 0.007 | 0.023 |
| Observations | 45,751 | 45,751 | 45,751 | 45,751 | 45,751 | 45,751 |

**Note:** Robust standard errors in parentheses, * p<0.05, ** p<0.01, *** p<0.001, models adjusted using population weights. Models include individual, household, area and asset controls, see table A 1. ATE – average treatment effect, ATT- average treatment effect on treated, ATU – average treatment effect on untreated, LATE – local average treatment effect, MPRTE – marginal policy relevant treatment effect. 1^st^ indicates an incremental shift in the instrument, 2^nd^ indicates an incremental shift in the absolute value of the propensity scores, and 3^rd^ indicates an incremental shift in the relative value of the propensity scores (see table 4)
